# Genome and secretome analysis of *Pochonia chlamydosporia* provide new insight into egg-parasitic mechanisms

**DOI:** 10.1038/s41598-018-19169-5

**Published:** 2018-01-18

**Authors:** Runmao Lin, Feifei Qin, Baoming Shen, Qianqian Shi, Chichuan Liu, Xi Zhang, Yang Jiao, Jun Lu, Yaoyao Gao, Marta Suarez-Fernandez, Federico Lopez-Moya, Luis Vicente Lopez-Llorca, Gang Wang, Zhenchuan Mao, Jian Ling, Yuhong Yang, Xinyue Cheng, Bingyan Xie

**Affiliations:** 1 College of Life Sciences, Beijing Normal University, Beijing, China; 2 Institute of Vegetables and Flowers, Chinese Academy of Agricultural Sciences, Beijing, China; 3 Laboratory of Plant Pathology, Department of Marine Sciences and Applied Biology, University of Alicante, Alicante, Spain; 4 Ministry of Education Key Laboratory for Biodiversity Science and Ecological Engineering, Beijing, China; 5 Key Laboratory of Biology and Genetic Improvement of Horticultural Crops, Ministry of Agriculture, Beijing, China

## Abstract

*Pochonia chlamydosporia* infects eggs and females of economically important plant-parasitic nematodes. The fungal isolates parasitizing different nematodes are genetically distinct. To understand their intraspecific genetic differentiation, parasitic mechanisms, and adaptive evolution, we assembled seven putative chromosomes of *P. chlamydosporia* strain 170 isolated from root-knot nematode eggs (~44 Mb, including 7.19% of transposable elements) and compared them with the genome of the strain 123 (~41 Mb) isolated from cereal cyst nematode. We focus on secretomes of the fungus, which play important roles in pathogenicity and fungus-host/environment interactions, and identified 1,750 secreted proteins, with a high proportion of carboxypeptidases, subtilisins, and chitinases. We analyzed the phylogenies of these genes and predicted new pathogenic molecules. By comparative transcriptome analysis, we found that secreted proteins involved in responses to nutrient stress are mainly comprised of proteases and glycoside hydrolases. Moreover, 32 secreted proteins undergoing positive selection and 71 duplicated gene pairs encoding secreted proteins are identified. Two duplicated pairs encoding secreted glycosyl hydrolases (GH30), which may be related to fungal endophytic process and lost in many insect-pathogenic fungi but exist in nematophagous fungi, are putatively acquired from bacteria by horizontal gene transfer. The results help understanding genetic origins and evolution of parasitism-related genes.

## Introduction

The fungus *Pochonia chlamydosporia* is a promising biological control agent for sedentary endoparasitic nematodes, including root-knot nematodes *Meloidogyne* spp., cyst nematodes *Heterodera* spp. and *Globodera* spp., and other plant parasitic nematodes (*Nacobbus* spp. and *Rotylenchulus* spp.)^1,2^. These nematodes cause dramatic economic losses in agricultural crops and some have been listed among the top 10 agronomically important nematodes^3^. In addition to conferring benefits via its nematophagous activity, which contributes to the suppression of plant-parasitic nematodes, recent studies show that *P. chlamydosporia* directly benefits plants, via the jasmonate signaling pathway^4,5^, by reducing flowering time, stimulating plant growth, and increasing seed production^5^. Therefore, *P. chlamydosporia* is a useful tool for protecting crops against plant endoparasitic nematodes and improving their agronomic performance.

In nature, the fungus *P. chlamydosporia* has a multitrophic lifestyle, which includes soil saprophytism, nematode egg parasitism, and plant root endophytism^1,6,7^, suggesting that *P. chlamydosporia* is able to evolve and adapt to various conditions and environments. In switching to different lifestyles and responding to different nutrition regimes, fungi transcribe divergent gene sets. Previous genomic and transcriptomic analyses of plant pathogens show distinct groups of genes linked to biotrophic, hemibiotrophic, and necrotrophic lifestyles^8^. The nematode endoparasitic fungus *Hirsutella minnesotensis*, with the capability to parasitize different nematode species including plant-parasitic and entomopathogenic nematodes, requires different signaling regulation mechanisms to respond to its various environments compared to nematode-trapping fungi^9^. Urea released by bacteria can trigger the nematode-trapping fungus *Arthrobotrys oligospora* to transition from the saprotrophic to the parasitic lifestyle^10^. The mechanisms of transition among the various fungal lifestyles are complex, reflected in the expression of different genes and signaling via distinct pathways, and most are still poorly understood. *P. chlamydosporia* treated with different nutritional stresses (resulting in saprophytism to true parasitism) shows marked transcriptional reprogramming between treatments, suggesting that special gene families and signal transduction events may be involved in multitrophic lifestyle transitions^1,11^. In particular, hydrolytic enzymes and transporters have been suggested to be related to fungal endophytic behavior^12^.

Secreted proteins play important roles in fungus-host and fungus-environment interactions, as well as in fungal pathogenicity. Plant pathogens use various genomic compartmentalization strategies to generate effectors to determine both their lifestyle and range of hosts^13^. Two secreted serine proteases (VCP1 and SCP1) and one secreted chitinase (PCCHI44) from *P. chlamydosporia* have been reported to be involved in nematode egg infection^14–16^. A recent study describes activity of chitin deacetylases (CDA1 and CDA2) together with chitosanases (CSN1-CSN9) as a determinant protein during nematode egg infection by *P. chlamydoporia*^17^. Transcriptome analysis shows that a large fraction of secreted proteins in the *P. chlamydosporia* strain 123 (PC123) genome are expressed during the endophytic process^12^, suggesting essential roles for secreted proteins in the multiple lifestyles of *P. chlamydosporia*. The whole genome sequence of PC123 isolated from the cereal cyst nematode *Heterodera avenae* has been published^12^, which provides useful information for further study of the parasitism mechanisms of the fungus. Analysis of CAZymes from PC123 genome reveals an expansion of glucosyl hydrolases mainly proteins related with degradation of chitin layer in the nematode egg. A highlighted expansion of chitosanases was identified in *P. chlamydosporia* instead entomopathogenic fungi *Metharhizium* spp. or mycoparasitic fungi *Trichoderma* spp. in a recent study^17^.

Distinct variants of *P. chlamydosporia* are associated with different host nematode species, and strains isolated from root-knot nematodes and cyst nematodes do not anastomose; they are considered different biotypes^1^. The sequence of the VCP1 subtilisin encoding gene can be used to identify the biotypes of *P. chlamydosporia*^18^. The genetic variations between isolates from cyst and root-knot nematodes were also examined by other molecular methods^19,20^ and demonstrated by virulence assay *in vitro*^21^. Nevertheless, the extent of genetic differentiation among the biotypes of the fungus is still poorly understood. Whole-genome investigation of the genetic differentiation among the biotypes is necessary for an in-depth study of the molecular mechanisms of fungal nematode parasitism, as well as the study of the adaptive evolution of the fungus. It is now possible to obtain a complete fungal genome by utilizing the single-molecule, real-time (SMRT) sequencing technology from Pacific Biosciences (PacBio) to generate long reads. SMRT is an innovative, potentially transformative, next-generation approach for determining DNA sequences, which can be combined with optical mapping technology to yield gapless, telomere-to-telomere genome assemblies^22,23^. SMRT provides the opportunity to obtain an accurate, complete *P. chlamydosporia* genome. In this study, we use a low-cost and efficient method, based on Illumina short reads and SMRT sequencing long reads, without optical mapping data, to acquire chromosome assemblies and a high-quality, complete genome of *P. chlamydosporia* strain 170 (PC170) isolated from the root-knot nematode *Meloidogyne incognita*, a distinct biotype from PC123. By comparative analysis of the genomes and secretomes of PC170 and PC123, we try to investigate the genetic differentiation between the two biotypes, identify parasitism-related secreted proteins, determine positively selected genes, and gene duplications, and explore the origin and evolution of parasitism-related genes in the fungus *P. chlamydosporia*. In addition, using transcriptome data from PC170 under different nutritional conditions, we try to investigate secreted proteins involved in responses to nutrient stress and adaptation. Our results will facilitate a deeper understanding of the genetic mechanisms of parasitism and adaptive evolution of the fungus *P. chlamydosporia*, and enable the effective development and utilization of this biocontrol agent.

## Results

### Assembly of the chromosome sequences of PC170

**Figure 1:**
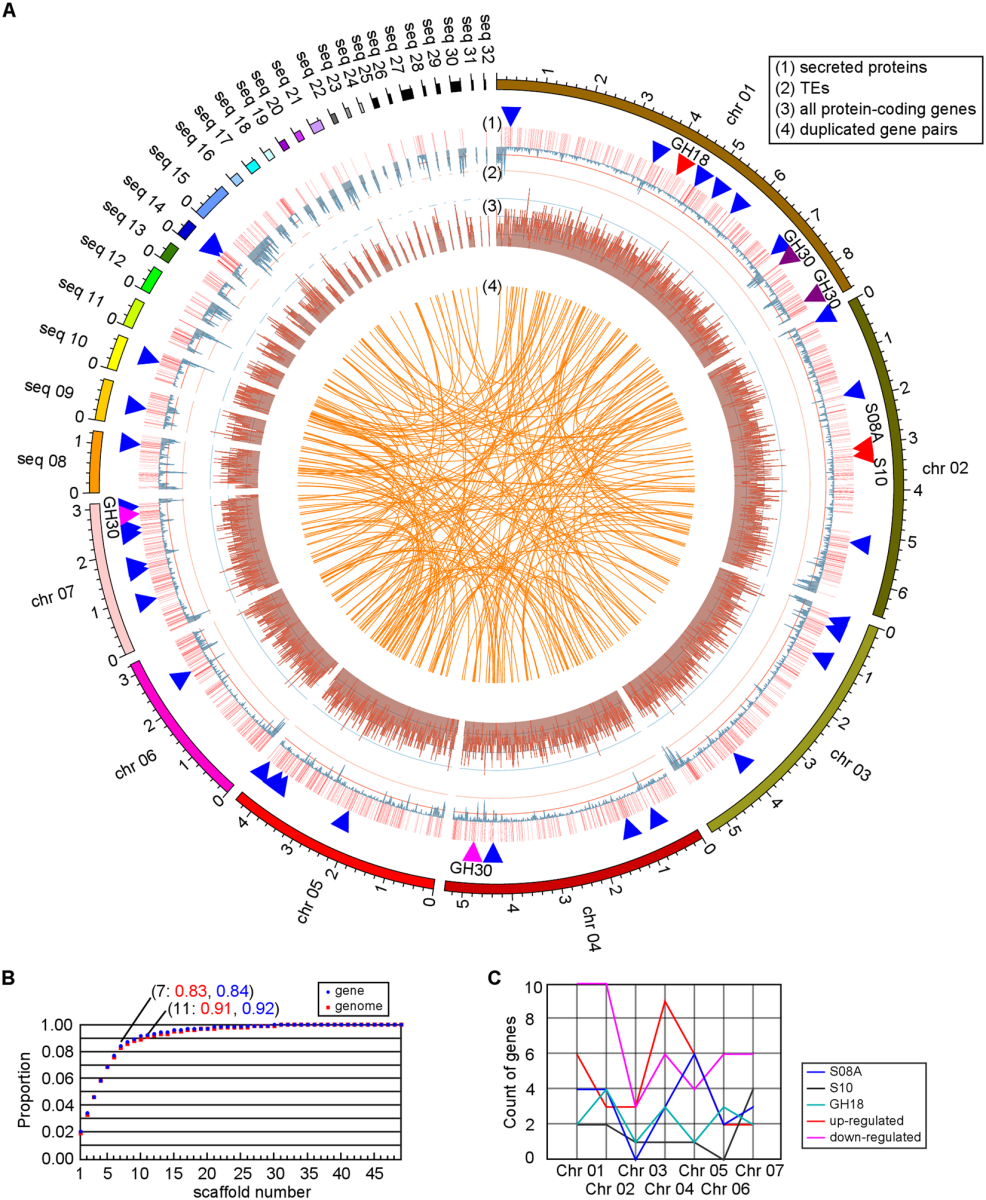
Chromosome sequences of *Pochonia chlamydosporia* strain 170 (PC170). (**A**) A map for seven chromosomes. The seven chromosomes (PCv3seq00001–00007) and other 25 long scaffolds (PCv3seq00008–000032, i.e., “seq 08”–“seq 32”) are shown, and other 17 short scaffolds with the sum length of 165,740 bp are not shown. The distribution of duplicated genes, all protein-coding genes, transposable elements (TEs), and secreted proteins are displayed. And three reported pathogenic genes (VFPPC_01099, GH18; VFPPC_03099, S10; VFPPC_03048, S08A), two duplicated GH30 gene pairs, and 32 positively selected genes are marked by red, pink/purple, and blue triangles, respectively. (**B**) The accumulation of gene components on PC170 scaffolds. The first seven scaffolds (PCv3seq 00001–00007) contain 83% of genomic sequences and 84% of predicted genes. (**C**) Chromosome distribution of S08A, S10, and GH18 genes, as well as up-regulated and down-regulated genes showed in Fig. 4.

Based on the Illumina and SMRT sequencing data (Supplementary Table S1), we obtain seven chromosome sequences for PC170 using five assembly steps (Table 1; Fig. 1A; Supplementary Figures S1–S3). We obtained 49 scaffolds (44.2 Mb) and identified 14,204 genes (Table 1; Supplementary Information). To check if chromosome sequences have been assembled, we analyzed the centromere proteins and telomere sequences of the scaffolds. In total, 11 centromere proteins are discovered, with 7 and 8, respectively, determined by Pfam annotation and homology-based methods by searching sequences against reported genes^24^ (Supplementary Table S2). Telomere sequences with high similarity to the *N. crassa* VR telomere region genomic sequences represented by TTAGGG tandem repeats^25^ are identified at the ends of 10 scaffolds by BLASTN alignments (Supplementary Table S3); they have also been found in the telomeric regions of Pezizomycotina fungi^26^. With Tandem Repeats Finder^27^, we determined TTAGGG repeats at the left ends of two scaffolds (PCv3seq00002 and PCv3seq00013) and the right ends of six scaffolds (PCv3seq00001, PCv3seq00004, PCv3seq00007, PCv3seq00010, PCv3seq00017, and PCv3seq00030) (Supplementary Table S4), indicating that the telomere regions of the chromosomes are involved. Considering the scaffold length, and the presence of centromere proteins and telomere sequences, we guess that seven assembled chromosome sequences are obtained in our dataset (PCv3seq00001–00007; Fig. 1A). These seven chromosomes comprise 83% of the assembled sequences and 84% of the predicted genes (Fig. 1B). The 32 larger scaffolds are shown in Fig. 1A, except for the 17 smaller scaffolds, which each contains less than 30 kb and has a total length of 165,740 bp, are not shown. We then mapped the genome sequence of PC123 onto the chromosomes of PC170 and found that 242 scaffolds of the PC123 genome sequence (34,183,092 bp, 81.43%) mapped to the seven chromosomes of PC170. Synteny analysis shows that, overall, 35,514,996 bp (84.60%) of the PC123 genome sequence matches 35,536,460 bp (80.37%) of the PC170 genome sequence with 96.45% identity (Supplementary Figure S4; Supplementary Table S5), with the high degree of similarity between the sequences of the two genomes.

**Table 1:** Five steps to obtain *Pochonia chlamydosporia* strain 170 chromosome sequences.

	Step 1	Step 2	Step 3	Step 4	Step 5	PC123^a^
Chromosomes	—	—	—	—	7	—
scaffold Number	184	114	100	70	49	894
scaffold Size (bp)	43,858,609	43,842,403	43,931,568	44,216,750	44,215,803	41,979,339
scaffold N50 (bp)	4,091,865	4,627,976	4,615,841	4,614,946	5,359,152	225,463
contig Number	631	548	316	114	114	8,512
contig Size (bp)	43,494,633	43,443,055	43,741,514	44,192,108	44,192,108	40,759,443
contig N50 (bp)	211,032	232,386	477,820	1,984,628	1,984,628	14,640
GC content (%)	49.5	49.5	49.5	49.5	49.5	49.9
Repeat sequences (%)	—	5.5	—	7.9	7.9	1.8
Gene Number	—	14,867	—	14,204	14,204	12,122^b^
Secreted Proteins	—	1,782	—	1,750	1,750	1,530
Small secreted proteins	—	743	—	728	728	654
SCS proteins^c^	—	153	—	157	157	116

Note: 1. Step 1: Obtained by Allpaths-LG assembly.

2. Step 2: Obtained by comparative analysis of Allpaths-LG assembly and SSPACE-LongRead assembly.

3. Step 3: Obtained by performing Jelly implemented in PBSuite based on Canu assembly.

4. Step 4: Obtained by performing Jelly based on the SMRT sequencing data that had been corrected by LoRDEC, and performing Pilon improvement and Tablet for manual correction.

5. Step 5: Obtained by comparative analysis of step4 assembled results and Canu assembly.

6. ^a^The genome sequence of *P. chlamydosporia* 123.

7. ^b^Among the 12,122 genes, 138 genes may be error predicted due to more stop codons found for one gene sequences, and these 138 genes were not used to predict secreted proteins.

8. ^c^SCS proteins, small cysteine-rich secreted proteins.

### Comparative analysis of transposable elements among genomes of invertebrate-pathogenic fungi in Hypocreales

**Figure 2:**
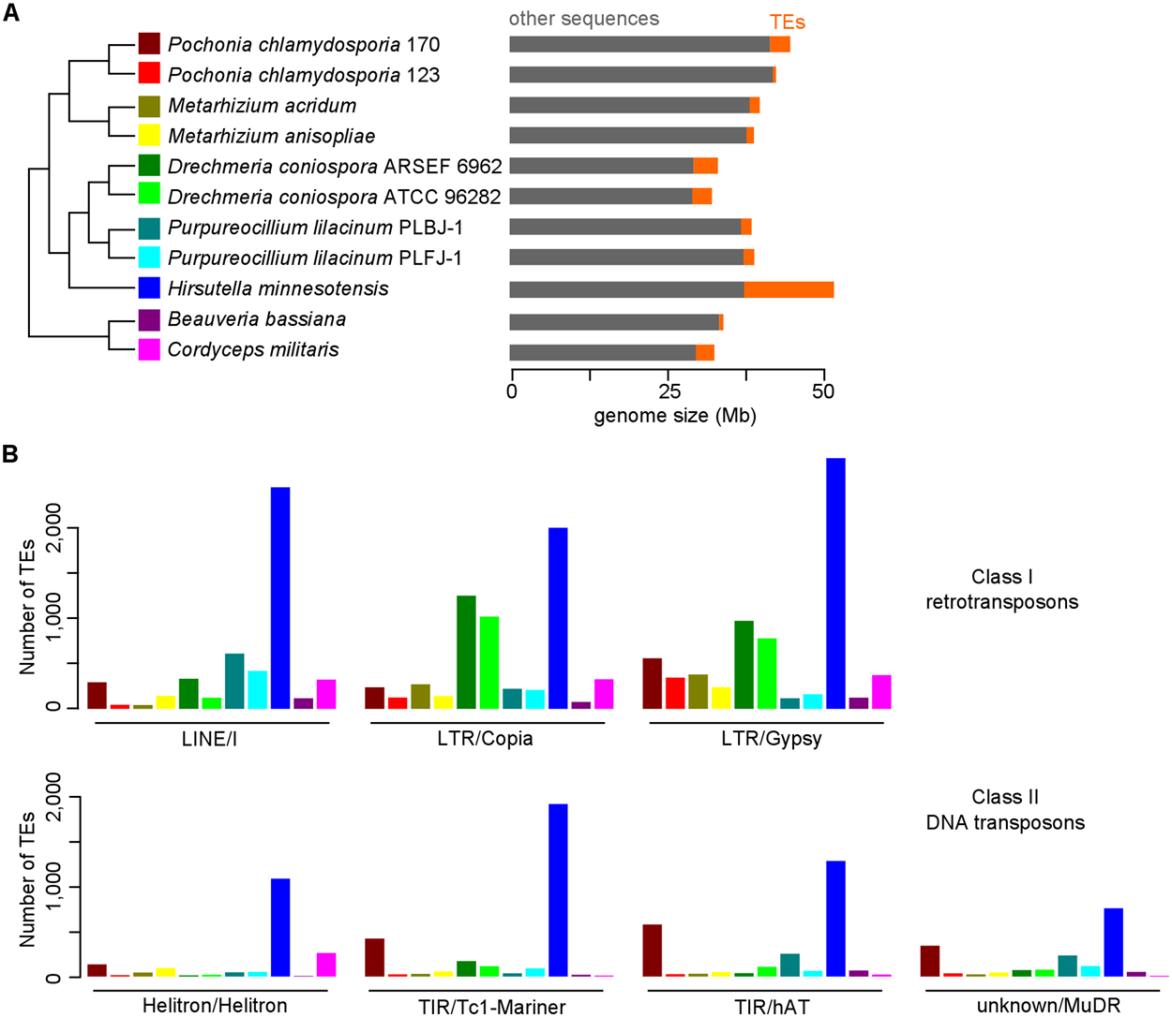
Comparative analysis of transposable elements (TEs) from 11 invertebrate-pathogenic fungi in Hypocreales. (**A**) Genomic contents of TEs in these fungal strains. TEs from the PC170, PC123, *M. acridum*, *M. anisopliae*, *D. coniospora* ARSEF 6962, *D. coniospora* ATCC 96282, *P. lilacinum* PLBJ-1, *P. lilacinum* PLFJ-1, *H. minnesotensis*, *B. bassiana*, and *C. militaris* genomes occupy about 7.19%, 1.21%, 4.03%, 3.07%, 11.64%, 9.70%, 4.37%, 4.33%, 27.68%, 1.94%, and 9.05%, respectively. The topology of phylogeny is indicated by previous studies^23,33,34,46,47^. (**B**) Distribution of retrotransposon and DNA transposon numbers in 11 fungal genomes.

Transposable elements (TEs) are instrumental for fungal genome evolution and are speculated to be involved in the evolution of pathogenesis^28^. Based on the genome sequences, we analyzed the TEs in PC170 and also reannotated TEs of PC123 (Supplementary Table S6). In PC170, 7.19% of sequences are identified as TEs and 0.67% as simple repeats and low complexity sequences. The abundant TEs belong to the Gypsy (Class I, retrotransposons), Tc1-mariner, hAT, and MuDR (Class II, DNA transposons) families, and also include unknown types. We find that more TEs are contained toward the ends of the scaffolds (Fig. 1A). We compared TE sequences among 11 invertebrate-pathogenic fungi in Hypocreales^9,12,23,29–34^ and found that TEs are rich in the genomes of the five fungal strains (PC170, two *Drechmeria coniospora* strains, *H. minnesotensis* and *Cordyceps militaris*) with a proportion of more than 7% genomic sequences (Fig. 2A). These sequences are mainly distributed in three retrotransposon types (I, Copia and Gypsy), four DNA transposon types (Helitron, Tc1-Mariner, hAT and MuDR) (Fig. 2B), and unclassified TEs (Supplementary Table S6). Many retrotransposons and DNA transposons are found in PC170 and *H. minnesotensis*, while a few kinds of DNA transposons are found in *D. coniospora* and *P. lilacinum*, indicating a dynamic evolution of TEs in invertebrate-pathogenic fungi.

### Secretome analysis and new parasitism-related gene prediction

**Figure 3:**
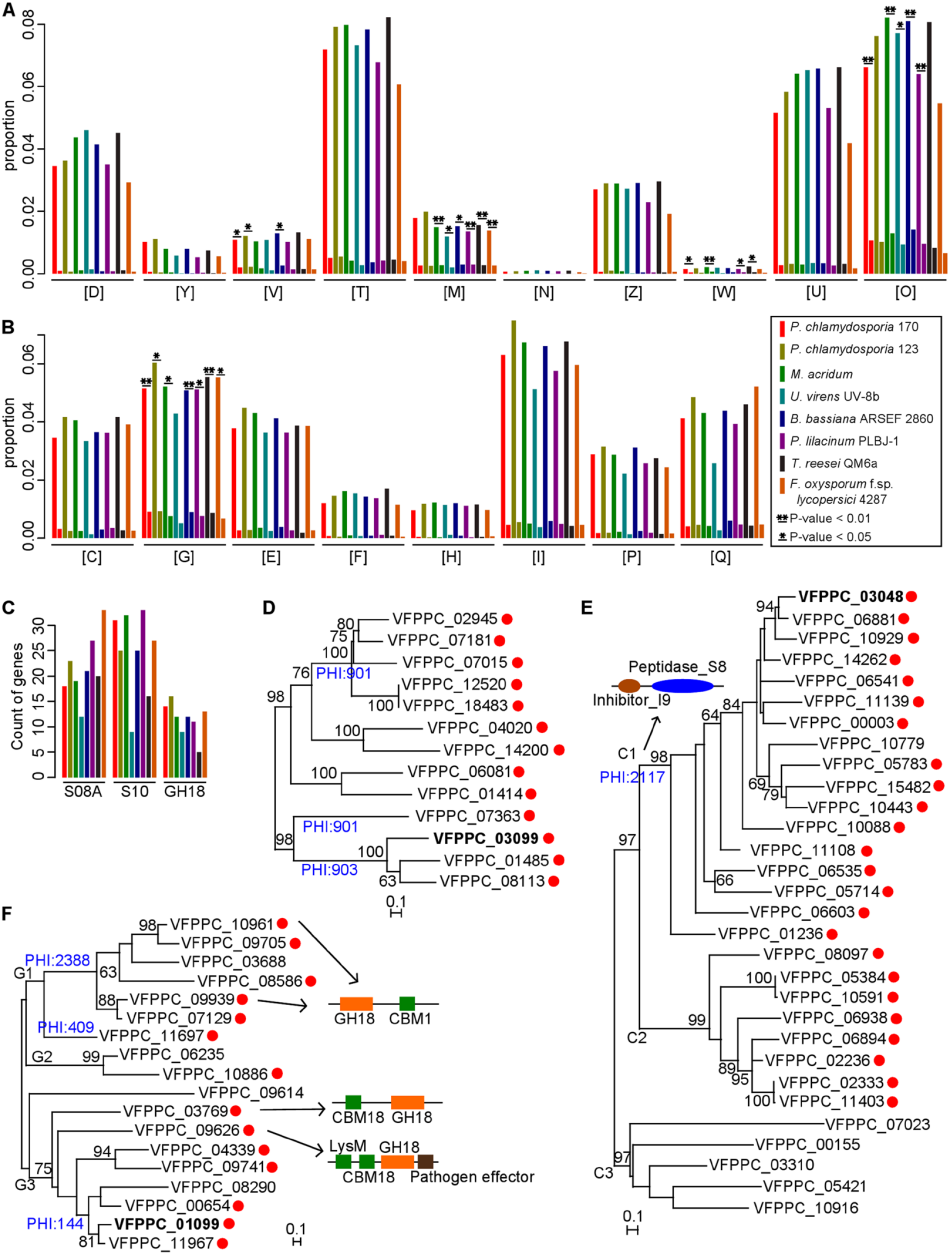
Analyses of secreted proteins. (**A,B**) Comparative analyses of secreted genes from eight fungal strains. All genes and secretomes of each strain are represented by two bars. These genes are annotated in “cellular processes and signaling functional” (**A**) and “metabolism” (**B**) categories of the euKaryotic Clusters of Orthologous Groups (KOG) database. Eighteen clusters in KOG annotations are shown: [D], cell cycle control, cell division, chromosome partitioning; [Y], nuclear structure; [V], defense mechanisms; [T], signal transduction mechanisms; [M], cell wall/membrane/envelope biogenesis; [N], cell motility; [Z], cytoskeleton; [W], extracellular structures; [U], intracellular trafficking, secretion, and vesicular transport; [O], posttranslational modification, protein turnover, chaperones; [C], energy production and conversion; [G], carbohydrate transport and metabolism; [E], amino acid transport and metabolism; [F], nucleotide transport and metabolism; [H], coenzyme transport and metabolism; [I], lipid transport and metabolism; [P], inorganic ion transport and metabolism; [Q], secondary metabolites biosynthesis, transport and catabolism. P-values from the binomial tests for comparison of the proportion of annotated secreted proteins/annotated proteins and the proportion of all secreted proteins/all proteins in each KOG cluster are shown. (**C**) List of genes belonging to protease S10, S08A, and CAZymes GH18 families. (**D**–**F**) Phylogenies of genes in S10, S08A, and GH18 families, respectively. Three reported pathogenic factors are marked in bold and secreted proteins are identified by red circles. The bootstrap values of larger than or equal to 60 are shown.

Secreted proteins are important for pathogenicity of parasitic fungi. In the genomes of PC170 and PC123, totally 1,750 and 1,530 sequences are identified to encode proteins with signal peptides but not transmembrane helices (Table 1; Supplementary Table S7). The distribution of the sequences of the putative secreted proteins on the chromosomes is shown in Fig. 1A. The KOG cluster analysis reveals three clusters (i.e., [O], post-translational modification, protein turnover, and chaperones; [G], carbohydrate transport and metabolism; and [R], general function prediction only) containing more than 100 secreted proteins in each cluster, which belong to three categories (i.e., cellular processes and signaling, metabolism, and poorly characterized) (Supplementary Table S8). Using the binomial test, we find that the proportion of annotated secreted proteins (PASG) in KOG clusters [O] (152/941, 16.15%) and [G] (129/735, 17.55%) is significantly higher than the proportion of secreted proteins (PSG) at the whole-genome level (1,750/14,204, 12.32%; P < 0.01) in PC170. However, in PC123, only the PASG in cluster [G] (110/713, 15.43%) is significantly greater than the whole-genome PSG (1,530/12,122, 12.62%; P < 0.05) (Fig. 3A,B; Supplementary Table S8). Proteins previously reported to be related to nematode infection are present in the two clusters, such as the serine carboxypeptidases (S10 family) and subtilisin serine proteases (S08A family) in cluster [O], and the chitinases (glycosyl hydrolase family 18, or GH18) in cluster [G].

We also compared the KOG annotations in different fungal species belonging to six Orders within Hypocreales (including plant pathogenic, endophytic, saprophytic, nematophagous and entomopathogenic fungi) (Supplementary Table S9). We found significant ratios between PASG and whole-genome PSG (the binomial test, P < 0.01) for proteins annotated within cluster [O] in three other fungi (*Metarhizium acridum*, *Beauveria bassiana*, and *Purpureocillium lilacinum*) (Fig. 3A) and within cluster [G] in two fungi (*B. bassiana* and *Trichoderma reesei*) (Fig. 3B). S08A family proteases and GH18 family chitinases are also common in these fungi (Fig. 3C; Supplementary Table S10, S11), but not in *Ustilaginoidea virens*, which has been reported to contain a reduced number of genes for polysaccharide degradation and secondary metabolism^35^.

We identified 14, 31, and 18 genes from S10, S08A, and GH18, respectively, in the PC170 genome (Fig. 3C; Supplementary Table S10, S11), most of which (13/14, 25/31, and 14/18, respectively) encode putative secreted proteins (Fig. 3D–F). We also identified 16 S10, 25 S08A, and 23 GH18 genes in the PC123 genome, which encode 8, 13, and 16 secreted proteins, respectively. Among them, homologs of SCP1 (VFPPC_03099, S10), VCP1 (VFPPC_03048, S08A), and PCCHI44 (VFPPC_01099, GH18), which are well-known fungal pathogenic proteins, are identified in genomes of the two *P. chlamydosporia* strains (Supplementary Table S12). However, functions of most other secreted proteins in the S10, S08A, and GH18 families are still unknown. Our previous study reported that, by knocking out the chitinase gene (PCCHI44, VFPPC 01099) and two protease genes (VFPPC 10088 and VFPPC 06535), the ability of the mutants to parasitize eggs of the root-knot nematode *M. incognita* is reduced, indicating the three genes have a partial role in the process of *P. chlamydosporia* infection of *M. incognita* eggs^36^.

We then analyzed the relationships among members in each of the three protein families (S10, S08A, and GH18). In the phylogeny of the S10 family (Fig. 3D), 9 of 13 secreted proteins are annotated PHI genes (PHI:901 and PHI:903), of which pathogenicity is unaffected after gene mutation. Two of the molecules (VFPPC_01485 and VFPPC_08113) are closest to SCP1 (VFPPC_03099), inferring that they might have similar functions to SCP1. The phylogenetic tree of the S08A family (Fig. 3E) comprises three major clades, and secreted proteins belong to two clades (C1 and C2). Notably, the 17 members of the C1 clade, including VCP1 (VFPPC_03048), have similar structures with peptidase_S8 and inhibitor_I9 domains (Pfam accession: PF05922), which are annotated by a PHI gene (PHI:2117), a reduced virulence gene after mutation, suggesting the possible existence of new pathogenic factors clustered on C1 clade. The phylogeny of the GH18 family (Fig. 3F) comprises three fungal chitinase clusters, as discovered in previous studies^37^: G1 contains some proteins with CBM1 modules at their C-terminal ends (VFPPC_10961 and VFPPC_09939), G2 contains proteins without a carbohydrate-binding module (CBM), and G3 contains some proteins with CBM18 modules (VFPPC_03769) and lysin motif (LysM; CBM50) modules (VFPPC_09626). The LysM effector in plant pathogenic fungi dampens the host response via chitin oligosaccharide sequestration^38^. Moreover, in addition to a LysM module, VFPPC_09626 also contains a pathogen effector domain (PF14856) (Fig. 3F), suggesting that it may be related to pathogenicity and worthy of further investigation. Moreover, the sequences of S10, S08A, and GH18 genes from closely related fungi (PC123, *M. acridum*, and *M. anisopliae*) are added into the phylogenetic tree of each group, and phylogenetic relationships are similar to that inferred from members of PC170 (Supplementary Figure S5). In addition, two other S08A genes from *Pochonia rubescens* are added to the phylogenetic tree and they show closer relationship to members of *Metarhizium* genes than to the PC170 genes, supporting the previous discovery^39^.

### Identification of proteins secreted in response to nutrient selection pressure

**Figure 4:**
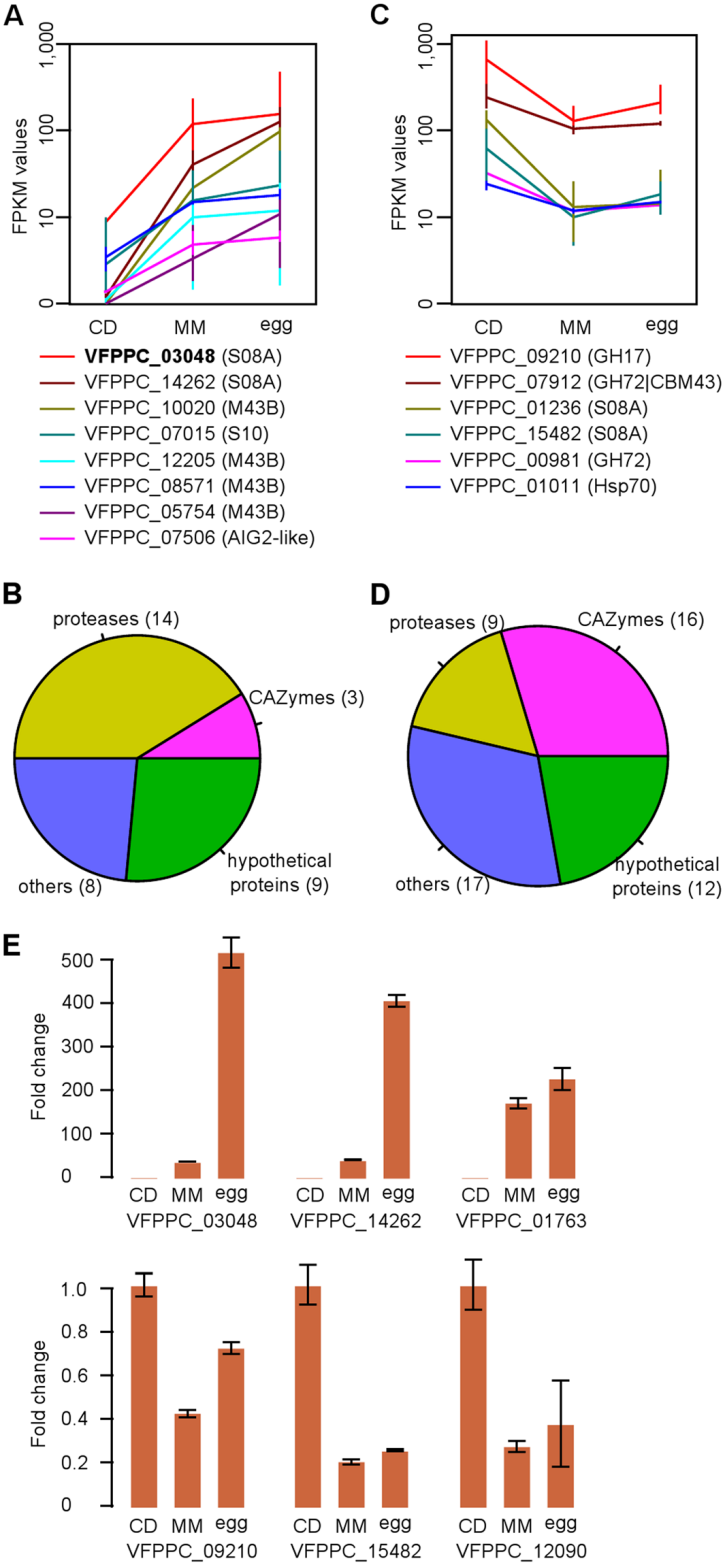
Transcriptome expression of secretomes responding to nutrient-selection pressure. (**A**) The transcriptome expressions of eight Pathogen-Host Interactions (PHI) database annotated genes. Transcriptome samples are prepared under three different nutrient conditions, including CD (nutrient rich medium samples), MM (minimal medium samples), and egg (minimal medium with root-knot nematode eggs samples). For these genes, the expression patterns of at least two-fold up-regulated between MM and CD, and up-regulated between MM-egg and MM are shown. A previously reported pathogenic factor (VFPPC_03048) is marked in bold. (**B**) A total of 34 secreted proteins representing the similar expression patterns as shown in (**A**). These genes include three CAZymes (GH2, GH127, and CE5) and 14 proteases (such as S08A, M43B, S10, M28E, S28, M36, M35, S53, and M20A). (**C**) The transcriptome expressions of six PHI database annotated gene. The expression patterns are of at least two-fold down-regulated between MM and CD, and up-regulated between MM-egg and MM. (**D**) A total of 54 secreted proteins representing the similar expression patterns as shown in (**C**). These genes include 16 CAZymes (such as GH16, GH17, GH72, GH25, GH37, GH63, GH132, GT31, GT90, CE4, CBM32, CBM43, AA5, and AA7) and nine proteases (such as S09X, A01A, S08A, G01, and S53). The detailed annotations for genes from (**B**) and (**D**) are shown in Supplementary Table S13. (**E**) RT-qPCR validation of six expressed genes. These genes are selected from (**B**) or (**D**). The results confirm the RNA-Seq data analysis. Bars represent the expression levels (fold change ± SE).

It has been reported that parasitic fungi behave differently in response to local nutrient availability^40^ and that some parasitism-related genes are differentially expressed in the saprotrophic-to-parasitic transition in *P. chlamydosporia*^11^. To further determine which secreted proteins are involved in the adaptation to nutrient sources and responses to nutrient stresses, we analyzed the transcriptome of the strain PC170 under three different nutrient conditions: Czapek Dox (CD) broth, a nutrient-rich medium; minimal medium (MM); and MM with root-knot nematode (*M. incognita*) eggs (MM-eggs) (Supplementary Information), prepared as described in a previous study^11^. We performed three biological replicates (R1, R2, and R3). Transcriptome analysis shows that 12,783 genes (90% of total) are expressed (RPKM >  = 0.5), including 1,492 secreted proteins (85.26% of total). We focus on the two major expression patterns for these genes, i.e., those distinctly up- or down-regulated between the CD and MM groups, but up-regulated in the MM-egg group compared to the MM group. We found 498 genes displaying obvious changes (>2-fold) with same trends in the 3 datasets (R1, R2, and R3) (Supplementary Table S13; Supplementary Figure S6; Fig. 4). Among them, 129 genes display the expression pattern of CD < MM < MM-eggs, i.e., they are up-regulated at least 2-fold in the MM compared to those in the CD group, and up-regulated in the MM-eggs group compared with those in the MM group (perhaps not to 2-fold in few cases), as shown in Fig. 4A. Among them, 34 genes encode secreted proteins, including 14 proteases, three carbohydrate-active enzymes, eight other proteins and nine functional unannotated genes (Fig. 4B; Supplementary Table S13). The proteases comprise six serine peptidases and eight mellopeptidases, including a well-known pathogenic factor, VCP1 (VFPPC_03048; S08A). Another PHI database-annotated S08A protease (VFPPC_14262), closely related to VFPPC_03048 in the phylogenetic analysis (Fig. 3E), is also identified (Fig. 4A). Only one PHI database-annotated S10 protease (VFPPC_07015) is found, but it is phylogenetically distant from the known pathogenic factor SCP1 (VFPPC_03099) (Fig. 3D). Among eight mellopeptidases, four PHI-annotated M43 peptidases are identified. A M36 protease (VFPPC_01763) is a homolog of the M36 protease (FVEG_13630) in *Fusarium verticillioides*, which cleaves maize chitinases^41^. Only one secreted M36 protease (VFPPC_01763 in PC170 and P123R_05288 in PC123) in each of the genomes is found, although another two non-secreted M36 proteases have previously been identified in PC123^12^. The three CAZymes include a cutinase (VFPPC_05424), a GH2 enzyme (VFPPC_14641), and a GH127 enzyme (VFPPC_07800). GH2 enzymes are involved in xylan and chitin degradation^42,43^, but the function of GH127 enzymes is not reported. Moreover, another eight proteins are annotated (Supplementary Table S13), including a PHI database-annotated AIG2-like protein (VFPPC_07506) and a FAD-binding protein (VFPPC_01275), the later with higher expression at the MM and MM-egg than CD conditions. Flavin adenine dinucleotide (FAD)-binding protein is reported to be involved in energy production, mycelial aggregation, and development in fungi^44^.

Moreover, 369 genes display another expression pattern of CD > MM < MM-eggs, i.e., expression of these genes are down-regulated at least 2-fold in the MM compared with those in the CD, but up-regulated in the MM-egg compared with those in the MM (Supplementary Table S13; Fig. 4C). Among them, 54 genes encode secreted proteins, including 16 CAZymes, nine proteases, 17 others and 12 unannotated genes (Fig. 4D). The gene families involved in this pattern are different from those in the former pattern; the CAZymes include two each GH16, GH17, and GH72 family members, and four other glycoside hydrolases, and the proteases include six serine proteases (three S09X, two S08, and one S53), two pepsin A enzymes, and one glutamic peptidase. Among other 17 annotated proteins, two carbohydrate-binding proteins (WSC domain-containing proteins), two common in several fungal extracellular membrane proteins (CFEMs), and two Hsp70 proteins are involved (Supplementary Table S13).

To validate the results of RNA-Seq data analysis, six genes representing these two expression patterns of CD < MM < MM-eggs and CD > MM < MM-eggs were selected to perform the real-time reverse transcription quantitative polymerase chain reaction (RT-qPCR) analysis. The results are shown in Fig. 4E. As expected, similar expression patterns of these genes are obtained by RT-qPCR and RNA-Seq analysis.

### Genes encoding secreted proteins under positive selection

Positive selection promotes the appearance of new phenotypes, thus playing a crucial role in evolution. Identification selected protein-coding genes under positive selection may contribute to understanding the interactions between fungi, their hosts, and their environments. In this paper, we compared the amino acid (aa) sequences of the putative secreted proteins in the two genome of *P. chlamydosporia* strains (PC170 and PC123) and determined that 32 genes encoding secreted proteins are under positive selection (dN/dS > 1) (Supplementary Table S14). Among them, 22 are functional unknown. The 10 annotated proteins include each of CAS1 appressorium specific protein (VFPPC_08840), chitosanase (GH75) (VFPPC_10847), cysteine-rich secretory protein (VFPPC_00045), FAD-binding domain-containing protein (VFPPC_10928), fungal calcium-binding protein (VFPPC_13067), WD domain-containing protein (VFPPC_07346), platelet-activating factor acetylhydrolase (VFPPC_14456), and scytalidoglutamic peptidase (G1) (VFPPC_09268), as well as two fungal hydrophobins (VFPPC_03630 and VFPPC_09461). Among them, CAS1 appressorium specific protein and chitosanase (GH75) are PHI-annotated proteins, and they may be related to the recognition and degradation of the host eggshells. The fungal calcium-binding protein may regulate signal transduction. These positively selected genes may play important roles in fungus-host interactions and the pathogenicity of the fungus *P. chlamydosporia* and await functional verification.

### Gene duplication of secreted proteins in the *P. chlamydosporia* genome

Gene duplication plays an important role in new gene origin and evolution, resulting in novel phenotypes and biological diversity^45^. Following the methods of gene duplication discovery in the previous studies (Materials and Methods), 248 duplicated gene pairs are identified in PC170 genome, of which, 165 have Pfam annotations and 22 have PHI annotations (Supplementary Table 15). Among them, 71 duplicated gene pairs (28.63%) are related to secreted proteins, including 21 pairs with only a single gene predicted to encode a secreted protein. Of the secreted gene pairs, 39 have Pfam annotations and 4 have PHI annotations, including cellulase (GH5) and GATA zinc finger. The proportion of duplicated genes in the secretome (8.11%, 142/1750) is higher than that in the whole genome (3.49%, 496/14204). To detect whether these duplicated genes obtain novel functions, we test their domain annotations and transcriptome expressions and find a weak correlation between the two duplicated genes (Pearson correlation coefficient <0.4) in 48.79% of the 248 gene pairs (11 of 23 differently annotated gene pairs, 43 of 83 unannotated gene pairs, and 67 of 142 gene pairs with the same annotation). This suggests sequence and functional divergence for these pairs. Notably, nearly 64.79% (46/71) of duplicated secreted gene pairs show a weak correlation (Supplementary Figure S7A; Supplementary Table S15). These duplicated gene pairs are putatively undergoing neofunctionalization.

### Origin and evolution of fungal GH30 genes

We find two duplicated gene pairs encoding secreted proteins from the GH30 family in the *P. chlamydosporia* genome; the first pair (VFPPC_07807-VFPPC_09315) has the same domain annotation and the other pair (VFPPC_02227-VFPPC_01957) has different annotations (Supplementary Table S15). The two duplicated genes in each pair are very weakly correlated (Pearson correlation coefficient <0.2), according to transcriptome expression data, indicating that they are undergoing neofunctionalization. We compare the protein sequences of the GH30 gene pairs and find low sequence identity in the aa alignments between the two pairs of genes, and their domains belong to different Pfam families (PF02055 for VFPPC_07807 and PF14587 for VFPPC_02227) (Supplementary Figure S8). These results indicate that the gene pairs are for two different proteins belonging to the GH30 family.

**Figure 5:**
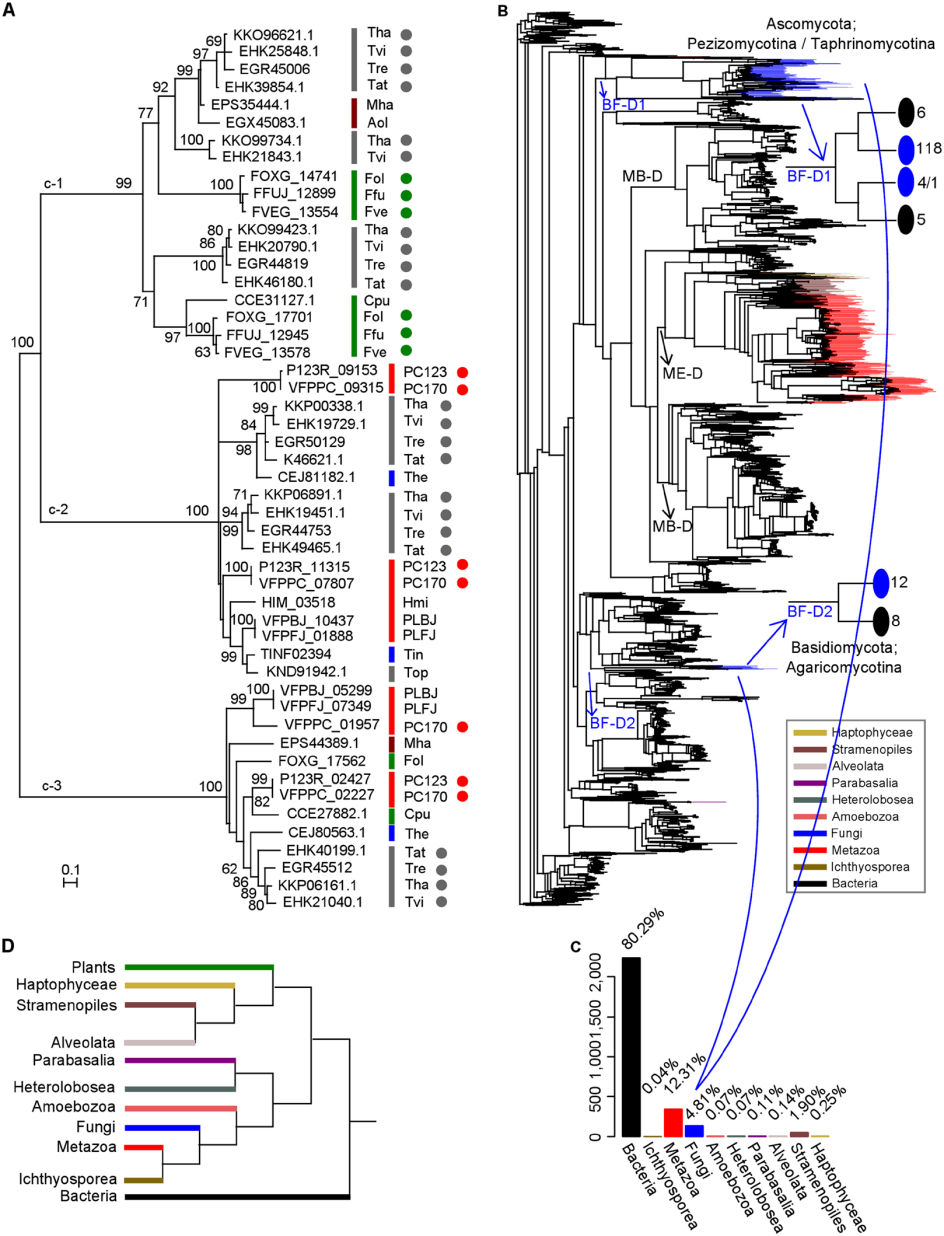
Detection of horizontal gene transfer (HGT) events for GH30 genes in fungi. (**A**) The Maximum likelihood phylogeny of 50 genes (bootstrap 1000) based on GH30 domain sequences annotated by Pfam PF14587 or PF02055 family. The best model of WAG + I + G + F was identified by ProtTest analysis. The first group (c-1) is comprised of plant-parasitic, endophytic, and mycoparasitic fungi (*Trichoderma* spp., *Fusarium* spp., and *C. purpurea*), and the two nematode-trapping fungi (*M. haptotylum* and *A. oligospora*). The two duplicated gene pairs of *P. chlamydosporia* are separately attributed to the other two groups (c-2 and c-3). The VFPPC_01957-VFPPC_02227 pair clusters with different lifestyle fungi (c-3), including plant parasites (*F. oxysporum* and *C. purpurea*), the nematode egg parasite *P. lilacinum*, the insect parasite *T. hemipterigena*, endophytic and mycoparasitic fungi (*Trichoderma* spp.), and the nematode-trapping fungus *M. haptotylum*. The VFPPC_07807-VFPPC_09315 pair clusters with nematode pathogens (*P. lilacinum* and *H. minnesotensis*), insect pathogens (*T. hemipterigena* and *T. inflatum*), and endoparasites and mycoparasites (*Trichoderma* spp. and *T. ophioglossoides*). (**B**) The phylogeny of 2,786 VFPPC_07807 homologous genes. The two clades (BF-D1 and BF-D2) contain Ascomycota and Basidiomycota genes, respectively. In BF-D1, 118 fungal genes are clustered with six bacteria genes, and another sub-clade contains four fungal genes and one bacteria gene. (**C**) Distribution of 2,786 genes, including 2,237 (80.29%) bacteria genes. (**D**) Phylogenetic topology of eukaryotes and bacteria. The topology is inferred from the global tree in study by Burki^88^ and topologies provided by Tree of Life Web Project (http://tolweb.org/tree/phylogeny.html).

We searched for homologs of the protein sequences encoded by the two gene pairs in gene sets from 37 fungal genomes (including 34 in Hypocreales) (Supplementary Table S9), and found homologous sequences in plant pathogens (*Fusarium oxysporum* and *Claviceps purpurea*), nematode pathogens (*P. lilacinum* and *H. minnesotensis*), insect-parasitic fungi (*Torrubiella hemipterigena* and *Tolypocladium inflatum*), and endophytic and mycoparasitic fungi (*Trichoderma* spp. and *Tolypocladium ophioglossoides*) (Supplementary Figure S8D). To our surprise, we did not find a homolog in the majority of the insect pathogens in Hypocreales, including the most closely related species (*Metarhizium* spp.)^12,46,47^. To confirm this result, we align the protein sequences of the two gene pairs against the NCBI nr database, which contains genomic data for multiple strains of *Metarhizium* and *Beauveria*, and again find no homologous sequences in any of these strains. However, homologs are found in nematode-trapping fungi (*Monacrosporium haptotylum* and *A. oligospora*). In total, 48 GH30 genes are found in Hypocreales fungi. Phylogenetic analysis of these homologs (including the two nematode-trapping fungi) shows that all GH30 proteins are clustered in three groups with more than 99% bootstrap support (Fig. 5A). The first group (c-1) is comprised of plant-parasitic, endophytic, and mycoparasitic fungi (*Trichoderma* spp., *Fusarium* spp., and *C. purpurea*), and the two nematode-trapping fungi (*M. haptotylum* and *A. oligospora*). The two duplicated gene pairs of *P. chlamydosporia* are separately attributed to the other two groups (c-2 and c-3). The VFPPC_01957-VFPPC_02227 pair clusters with different lifestyle fungi (c-3), including plant parasites (*F. oxysporum* and *C. purpurea*), the nematode egg parasite *P. lilacinum*, the insect parasite *T. hemipterigena*, endophytic and mycoparasitic fungi (*Trichoderma* spp.), and the nematode-trapping fungus *M. haptotylum*. Only one gene is found in each fungal genome, except in the genome of the fungus *P. chlamydosporia*. The VFPPC_07807-VFPPC_09315 pair clusters with nematode pathogens (*P. lilacinum* and *H. minnesotensis*), insect pathogens (*T. hemipterigena* and *T. inflatum*), and endoparasites and mycoparasites (*Trichoderma* spp. and *T. ophioglossoides*). Interestingly, this gene is also duplicated in *Trichoderma* fungi.

To further explore the origin and evolution of the two duplicated gene pairs of the GH30 family, we deposited the aa sequences of the two PC170 genes (VFPPC_07807 and VEFFC_02227) in the NCBI nr database (E-value threshold of 1e-5) for alignment analysis. For VFPPC_07807-VFPPC_09315 pair, 2,786 PF02055 domains are selected for phylogenetic analysis (Fig. 5B,C; Supplementary Table S16). The phylogenetic topology reveals that all eukaryotic domain branches are surrounded by prokaryotic domain branches (Fig. 5B). All fungal domains cluster into 3 clades, with 118 (Ascomycota), 4 (Ascomycota) (Supplementary Figure S9), and 12 (Basidiomycota) domains, and they are separated by bacterial clades (Fig. 5B). Fungi in Ascomycota and Basidiomycota are separately clustered to form two large groups (BF-D1 and BF-D2), indicating their disparate origins. In the Ascomycota group (BF-D1), all Ascomycota fungi (except four from three classes) gather to form a monophyletic group, then gather with a clade of bacteria mainly comprising the actinomycetes (Actinobacteria) (five of six), indicating a close relationship between the GH30 genes in Ascomycota fungi and in Actinomycetales bacteria. These Ascomycota fungi are divided into four subgroups (Supplementary Figure S9), and each group includes fungi belonging to different Classes and Orders. The phylogenetic relationship of the GH30 genes could not reflect the lineages of the fungal species. The two duplicated genes (VFPPC_07807 and VFPPC_09315) in *P. chlamydosporia* are clustered into a clade with some Hypocreales fungi, with VFPPC_07807 (accession number: OAQ66228) clustering close to endophytic *Trichoderma* spp. and VFPPC_09315 (accession number: OAQ61486) clustering close to nematode pathogens (*H. minnesotensis*) (Supplementary Figure S9). Moreover, the other four Ascomycota fungi cluster to a clade with other bacteria, but closest to a bacterium in Actinomycetales (*Actinospica robiniae*). Most of the eukaryotic domains cluster to a clade that is parallel with the bacterial clade (MB-D) (Fig. 5B).

For the VEFFC_02227-VFPPC_01957 gene pair, 689 homologs with a PF14587 domain are used to construct a phylogenetic tree (Supplementary Figure S10; Supplementary Table S17). All fungal sequences gather in a large cluster, embraced by bacterial branches, indicating that they are also acquired from bacteria. Within the fungal cluster, complex relationships are observed among the fungi, making it difficult to display the lineages of species. The two duplicated genes (VEFFC_02227 and VFPPC_01957) are clustered in clades with distant relationships to fungi, indicating that this duplication event may have happened early, followed by convergent evolution after neofunctionalization.

## Discussion

Whole-genome sequences are the fundamental data source for gene identification and functional annotation, as well as comparative analysis. More than 1,900 fungal genomes have been reported (https://www.ncbi.nlm.nih.gov/genome/browse/; 2016-10-30), but most of them contain hundreds or thousands of fragment sequences and lack chromosome information. Recently, the complete genomes of two fungi are obtained by hybrid assembly methods based on Illumina high-throughput sequencing, SMRT sequencing, and optical mapping data^22,23^. Data generated by SMRT sequencing technology enable the resolution of complex regions, including repetitive DNA and insertions, in diploid species^48^. In addition to the development of assembly algorithms, the technology makes it possible to address the difficulties in diploid or polyploid genome assembly, enabling high-quality genome sequences to be acquired^49^. In this study, we assemble the chromosome sequences of the haploid fungus PC170 (44.2 Mb) using a low-cost and efficient method based on three libraries of Illumina sequencing reads (~210×) and SMRT sequencing reads (~32×) (Supplementary Table S1), but without optical mapping data, distinguishing it from previous studies^22,23^. Seven chromosome sequences are obtained, which included 84% of predicted genes. We suggest that this method may be applicable for the assembly of other fungal chromosomes.

Compared with the published genome of PC123^12^, we find the proportion of TEs in PC170 notably higher (7.19% in PC170 vs. 0.46% in PC123)^12^. We then reannotate TEs in PC123 genome with the same method and acquire TEs with 1.2% of genome sequences (Supplementary Table S6). We suggest that the differences of TEs between the two genomes mainly originated from different sequencing and assembly methods. The new methods can provide genomic regions that are previously not assembled or poorly assembled, including regions that are populated by repetitive sequences, such as transposons^22^. For the same reason, only 84.60% of the PC123 genome sequence could be mapped to 80.37% of the PC170 genome sequence by syntenic analysis, but, the identity of the sequences is more than 96.45%, indicating a high level of sequence similarity and conserved synteny between the two biotype genomes (Supplementary Figure S4). However, the authentic differences existing between the two biotypes are detected. We compared proteases predicted in the PC170 and PC123 genomes, including 525 and 514 genes, respectively (Supplementary Table S10). Their differences are mainly in the S08A (31 vs. 25), S09X (63 vs. 68), S10 (14 vs. 16), M43B (18 vs. 14), C14B (3 vs. 6), and I09 (5 vs. 2) families. The subtilisin (S08A) and carboxypeptidase (S10) enzymes may be involved in host recognition and pathogenicity, like the well-known VCP1 and SCP1, which are related to nematode egg penetration^14,15,17^. An overexpression of VCP1 and SCP1 under stress increases fungal capabilities to parasitize nematode eggs^17^. Metacaspase Yca1 (C14B) in *Saccharomyces cerevisiae* contributes to the fitness and adaptability of growing yeast through an aggregate remodeling activity^50^. Other peptidases, such as serine peptidase (S09) and pappalysin-1 (M43B), as well as peptidase B inhibitor-containing inhibitors (I09) of subtilisin serine peptidases, may be related to the growth and development of the fungus. We also find fewer chitinases in the PC170 genome (18 genes) than in the PC123 genome (23 genes). Chitinases belonging to the GH18 family catalyze the hydrolysis of beta-1,4-linkages in chitins, which are the main components of invertebrate exoskeletons, nematode eggshells, and fungal cell walls^51,52^. The known biological functions of fungal chitinases include the decomposition of exogenous chitin and the degradation and remodeling of fungal cell walls^51,52^. The differential and abundant proteases and chitinases in *P. chlamydosporia* genome may contribute to adaptation to a broad array of hosts and environments, as well as pathogenicity. Therefore, we speculate that the divergences between the two biotype genomes might reflect the evolution of *P. chlamydosporia* for long-term adaptation to diverse hosts and environments. Moreover, except the well-known pathogenicity-related genes (VCP1, SCP1, and PCCHI44), some new pathogenic factors are predicted in the fungal genome in this paper, given that they share similar functional domains with the three well-known pathogenic proteins, could be annotated by PHI database and are putative secreted proteins, such as VFPPC_01485 and VFPPC_08113, which are close to SCP1 (VFPPC_03099), and so on. VFPPC_09626 contains a pathogen effector domain (PF14856) and a LysM module (Fig. 3F), suggesting that it is related to the fungal pathogenicity.

Genetic reprogramming, which reflects the adaptive processes resulting from fungus–host interactions, has been observed in nematode- and insect-parasitic species. The fungus *P. chlamydosporia* has multitrophic lifestyles. Previous studies on *P. chlamydosporia* showed that gene expression profiles changed under different nutritional conditions^11^ and thousands of gene expressed at endophytic phase^12^. In our study, we compared transcriptomes of PC170 at three different trophic status, CD (nutrient-rich medium), which is predicted to repress fungal parasitism, MM (nutrient-poor liquid medium), which is predicted to enable de-repression of genes associated with parasitism, and MM-eggs (host inducing)^11^. We focused on the two major patterns of gene expression, CD < MM < MM-eggs and CD > MM < MM-eggs. We identified 34 genes encoding secreted proteins that display the first pattern, and half of them are proteases (mainly serine peptidases and mellopeptidases) and CAZymes. Some pathogenic factors are involved, such as VCP1 and PHI-annotated proteases. We guess these genes would contribute to lifestyle switching from saprophytism to parasitism. We also identified 54 genes encoding secreted proteins displaying the second expression pattern, which including more CAZymes and less proteases compared to the first pattern. We found that most of gene families involved in the two patterns are different. Some genes perhaps are functionally important, such as CFEM domain-containing protein, which can influence pathogenic fungal adhesion by enabling recognition of and adherence to a host^53^; SUN protein (GH132), which is involved in the fungal morphogenetic processes of cell wall biogenesis and septation^54^, may contribute to fungal growth and adaptation to the environment; and WSC domain-containing protein, which is required for the maintenance of cell wall integrity and for stress responses^55^. We also compared these genes involved in nutritional transitions in PC170 with those involved in endophytic phase in PC123^12^, and found that some secreted proteins may be involved in multiple lifestyle transitions, including peptidases (such as serine peptidases: S08A, S09X, S10, S53; metalloproteinase: M20A, M36, M28; and aspartic endopeptidase: A01A), CAZymes (such as glycoside hydrolases: GH16, GH17, GH25, GH72; glycosyltransferase: GT31; acetyl xylanesterase: CE5; and glyoxal oxidase: AA5), and some others (such as heat shock protein: Hsp70, CFEM domain-containing protein, WSC domain-containing protein, FAD binding domain-containing protein). These genes encoded by *P. chlamydosporia* may contribute to its ecological niche as a multiple lifestyle fungus. Moreover, to explore the relationships of TEs with the genes related to pathogenesis in *P. chlamydosporia*, we examined the TE expressions at the whole-genome level (Supplementary Figure S11) based on alignments of RNA-Seq data under three nutrient conditions (including three replicates). We identified 13 TE clusters with different expression levels between CD and MM/MM-eggs nutrient conditions. Meanwhile, we checked the distribution of protein-coding genes within the range of 5000 bp flanking sequences of these TEs. We found that eight secreted proteins (each of GH5, GH76, S08A, S33, neutral/alkaline non-lysosomal ceramidase, calcineurin-like phosphoesterase, FAD binding protein, and hypothetical protein) and five PHI database annotated genes (each of S08A, ABC transporter, autophagy protein, E1-E2 ATPase, and MAD3/BUB1 homology region gene) are involved in these regions, however, few co-expression relationships are observed between TEs and physically clustered genes obviously (Supplementary Table S18). Therefore, based on our current results, it is difficult to presume the relationship of TEs with the genes related to pathogenesis in *P. chlamydosporia*.

Gene duplication, HGT, novel function acquisition, and positive selection are important mechanisms that shape genetic origin and evolution. In the *P. chlamydosporia* genome, we identify 248 duplicated gene pairs; of those, 71 pairs encode secreted proteins, including cellulase (GH5), GH30 proteins, and copper/zinc superoxide dismutase (Supplementary Table S15), which may be important for the parasitism and adaptation of the fungus. Of them, two duplicated gene pairs encoding secreted GH30 O-glycosyl hydrolases are found; however, they are absent in the great majority of Hypocreales fungi, but their homologs are found in the nematode pathogens (*P. lilacinum* and *H. minnesotensis*) and the endophytic and mycoparasitic fungi (*Trichoderma* spp.). Phylogenetic analysis shows that these genes in Ascomycota fungi might originate from bacteria by HGT (Fig. 5B; Supplementary Figure S10; Supplementary Table S16,S17). GH30 was reported to be related to plant cell wall degradation^43,56^ and GH30 gene was expressed in barley root colonization in PC123^12^. We suppose these GH30 proteins in *P. chlamydosporia* might be related to the fungal endophytic lifestyle. Moreover, we identify 32 genes under positive selection by comparing the genomes of PC170 and PC123 (Supplementary Table S14). Although most of those genes are of unknown function, we believe that these positively selected genes are important for the parasitism and adaptation of the fungus *P. chlamydosporia* based on the functions of 10 annotated genes, such as CAS1 appressorium specific protein, which may be related to host infection. The chitosanase (GH75) may play an important role in regulating the multitrophic lifestyles of *P. chlamydosporia*, as it could be involved in chitin metabolism, which is required for endophytic and saprophytic lifestyles, and it is expressed during nematode egg infection^17^. Genes homologous to VFPPC_00045 (the cysteine-rich secretory protein family) are found in *Metarhizium* spp. (such as MAC_00975 in *M. acridum*)^29,32^ and the plant pathogen *U. virens* (NCBI Accession: KDB15609)^35^. Genes that are previously reported to belong to the cysteine-rich secretory protein family are found to be associated with host adaptation or specialization^57^. FAD-binding protein is reportedly involved in fungal growth^44^. Positive selection in fungi may result in high variability and adaptability of the organisms during evolution.

## Materials and Methods

### Fungal strain

PC170 was originally isolated from the eggs of root-knot nematode *M. incognita*. It had been deposited into the China General Microbiological Culture Collection Center (CGMCC, number 8860)^46^. Previously, the biotypes of *P. chlamydosporia* were identified by molecular methods^18–20^ and *in vitro* assays that were also confirm by the detection of VCP1 gene sequences^21^. The variant sites of amino acid (aa) sequences of the VCP1 genes (subtilisins)^18^ include two characteristic sites 171 and 208 (“E” and “G” in VCP1 of root-knot nematode isolates, and “Q” and “A” in VCP1 of cyst nematode isolates). For PC170, its infection ability to *M. incognita* eggs had been previously tested^36^ and its previously sequenced (by PCR technology) beta-tubulin sequences (the same sequences of VFPPC_01610 gene in the assembled genome, from 953 bp to 1188 bp) are the most similar to the deposited AJ012713 sequences in NCBI collected data, which was from the *P. chlamydosporia* strain Vc10 isolated from *M. incognita* eggs in UK^58^.

### Genome sequence assembly

We assembled the chromosome sequences of PC170 in five steps. Step 1: We obtained a draft genome sequence of 184 scaffolds (631 contigs) using Allpaths-LG based on three libraries of Illumina HiSeq 2000 sequencing reads by BGI-Shenzhen (China)^59^. Step 2: For four SMRT cells of PacBio RS long reads (Supplementary Table S1), we performed error correction analysis using LoRDEC^60^, and these corrected reads were used for the following analyses. We used SSPACE-LongRead^61^ to build scaffolds for Allpaths-LG-assembled contigs. The conflicting scaffolds between Allpaths-LG and SSPACE-LongRead methods were resolved (Supplementary Figure 1), which yielded 114 scaffolds. Step 3: The corrected RS long reads were *de novo* assembled using Canu^49^ and the assembled sequences were improved by Pilon^62^. These sequences showed high synteny compared to the Step 2 results (Supplementary Figure 2) and were split into units of 100 kb, which were used to further improve the Step 2 assembly using Jelly^63^, yielding 103 scaffolds. Step 4: We used used Tablet^64^, a graphical viewer for Illumina read alignments, to manually correct the assembled sequences (Supplementary Figure 3A). Seventy scaffolds were obtained, and these sequences showed high syntenic blocks against sequences from Step 2 results (Supplementary Figure 3B), but improved the sequence assembly. Step 5: A comparison analysis of the sequences from the Step 4 results and the Canu assembly indicated that 35 scaffolds could be further improved (Supplementary Figure 4). Finally, we obtained 49 scaffolds for PC170 containing 7.85% repeat sequences and 14,204 predicted protein-coding genes (Table 1; Supplementary Information). Then, we detected 11 centromere proteins by Pfam annotation and homology-based methods via searching the sequences against reported centromeres in other filamentous fungi^24^ (Fig. 1A; Supplementary Table S2). In addition, we identified telomere sequences at the ends of 10 scaffolds by BLASTN alignment against *Neurospora crassa* VR telomere sequences (NCBI accession: M37064.1; Fig. 1A; Supplementary Table S3)^25^. Our analysis with Tandem Repeats Finder^27^ revealed TTAGGG repeats at the ends of eight scaffolds (Supplementary Table S4). In the end, we hypothesized that our assembly method obtained seven chromosome sequences (PCv3seq00001–00007; Fig. 1A; details in Supplementary Information) and drew the genomic map using Circos^65^.

### Detection and annotation of secretomes

We identified secreted proteins as described previously^33,66^, by detecting proteins with signal peptide sequences but without transmembrane spans. The signal peptides were determined by SignalP, version 4.0^67^; TargetP, version 1.1^68^; Phobius, version 101^69^; and Predisi algorithms^70^, and transmembrane spans were identified by SignalP, Phobius, and TMHMM, version 2.0c^71^. We found 1,750 (12.32%) genes in PC170 that contained signal peptides (supported by at least two algorithms) and no transmembrane sequences (supported by at least one algorithm). With the same method, we identified 1,530 secreted proteins in PC123 gene sets, although Larriba *et al*. predicted 2,485 secreted proteins with the same gene sets using a single SignalP algorithm^12^. Moreover, we performed the EuKaryotic Orthologous Groups (KOG)^72^ and Pathogen-Host Interactions (PHI) database^73^ annotation analyses for secretomes using BLASTP with an E-value cut-off of 1e-5 and 1e-50, respectively. Some secreted proteins were annotated as CAZymes or proteases, which was confirmed by uploading protein sequences to three web servers for annotation, including CAT^74^ and dbCAN^75^ for CAZymes annotation, and MEROPS^76^ for protease annotation. Genes in other species (Supplementary Table S9) were analyzed using the same methods for comparative analyses.

To investigate gene duplication in *P. chlamydosporia*, we employ the orthogroup inference method and the bidirectional best hit method^77,78^ based on gene sequences from seven fungal species (Supplementary Information), which indicates orthologs and paralogs information for genes from whole-genome sequences. Following the methods of gene duplication discovery in the previous study^45^, we manually confirm the paralogous genes in PC170 and identify 248 duplicated gene pairs.

### Phylogenetic analysis

To perform phylogenetic analyses for the aa sequences of genes in the protease S10 and S08A families, and the CAZymes GH18 family, we obtained their domain sequences (Peptidase_S10: Serine carboxypeptidase; Peptidase_S8: Subtilase; Glyco_hydro_18: GH18) from the Pfam database, and performed sequence alignment using MUSCLE, version 3.8.31^79^; we also investigated the best phylogenetic models (WAG + I + G for S10 and S08A genes; WAG + I + G + F for GH18 genes) using ProtTest, version 3.4 (Supplementary Information)^80^. Finally, we built the maximum-likelihood trees for these genes using Mega, version 6.06^81^ and PhyML, version 3.1^82^, with a bootstrap value of 1000.

To analyze the phylogenetic relationships in the GH30 protein family, we deposited the aa sequence from the PC170 gene (VFPPC_07807) in the NCBI nr database (E-value threshold of 1e-5) for alignment analysis, and found 3,753 homologous sequences, including 2,534 (67.52%) genes from bacteria, 1,210 (32.24%) genes from eukaryotes, and 9 genes (0.24%) collected in the Protein Data Bank. Their length distribution ranged from 90 to 2,713 aa, with the majority at 437 to 542 aa (2,679, 71.38%) (Supplementary Figure S7B). Most of the sequences (3,747 of 3,753) encode PF02055 domains (GH30 family) ranging from 47 to 592 aa, with seven gene sequences from Metazoa encoding two domains each and one gene sequence from Metazoa encoding three domains. To avoid the presence of too many gaps for alignment, we used 2,786 GH30 domain (Pfam accession: PF02055) sequences (from 360 to 480 aa) to build phylogeny, including 2,237 (80.29%) from bacteria, 549 (19.70%) from eukaryotes, and 2 domains from 1 protein (KNC29753.1) of *Lucilia cuprina* (Metazoa) (Fig. 5B,C; Supplementary Table S16). We also deposited the aa sequence of the PC170 gene (VFPPC_01957) in the NCBI nr database (E-value threshold of 1e-5) for alignment analysis, and found 1,651 homologous sequences, including 1,307 (79.16%) genes from bacteria and 344 (20.84%) genes from eukaryotes. Their lengths ranged from 90 to 2,924 aa, with the majority from 430 to 528 aa (944, 57.18%) (Supplementary Figure S7C). Most of the sequences (933) encoded PF14587 domains (GH30) with lengths ranging from 84 to 391 aa. To avoid the presence of too many gaps for alignment, we used 689 GH30 domain (Pfam accession: PF14587) sequences ranging from 180 to 260 aa to build phylogeny, including 562 (81.57%) from bacteria and 127 (18.43%) from eukaryotes (Supplementary Figure S10; Supplementary Table S17). Sequence alignments were performed by MUSCLE. As building a large phylogeny for thousands of genes is challenging, we used FastTree, version 2.1.9^83^, with the JTT model to analyze the data.

### Discovery of positively selected genes

For each pair of orthologous genes in PC123 and PC170, we investigated the positive selection signal (dN/dS > 1) using CODEML (with M0 model) implemented in PAML, version 4.8^84^. For the 32 positively selected secreted genes, we then performed DnaSP, version 5.10.1^85^ and KaKs_Calculator 2.0^86^ to calculate the dN/dS values, and these results supported the CODEML calculations.

### Transcriptome preparation and analysis

The PC170 strain was grown on potato dextrose agar. The mycelia were harvested and prepared for transcriptome sequencing treatments, with three biological replicates for each group: nutrient-rich CD (30 g⋅l^−1^ sucrose, 3 g⋅l^−1^ NaNO_3_, 0.5 g⋅l^−1^ MgSO_4_, 0.5 g⋅l^−1^ KCl, 1.0 g⋅l^−1^ K_2_HPO_4_, and 0.01 g⋅l^−1^ FeSO_4_), which was predicted to repress parasitism; nutrient-poor liquid MM (1 mg⋅l^−1^ sucrose, 14 mg⋅l^−1^ NaNO_3_, 0.25 g⋅l^−1^ MgSO_4_, 0.25 g⋅l^−1^ KCl, 0.5 g⋅l^−1^ K_2_HPO_4_, and 0.06 g⋅l^−1^ FeSO_4_), which was predicted to de-repress genes associated with parasitism; and MM-egg, which was prepared to induce parasitism, as described in a previous study^11^. RNA isolation was carried out using the RNeasy Plant Mini Kit (Qiagen), according to the manufacturer’s instructions. The total RNA was sequenced using Illumina HiSeq 2000 at Berry Genomics (Beijing, China). The values for the expressed fragments per kilobase of transcript per million mapped fragments of genes were obtained by performing analysis referring from reported workflow (details in Supplementary Information)^87^. The gene expression was also validated by RT-qPCR. The details and primer sequences were shown in Supplementary Information and Supplementary Table S19, respectively.

### Accession numbers

The genome sequences have been deposited at DDBJ/ENA/GenBank under the accession LSBJ00000000. The version described in this paper is version LSBJ02000000. And the RNA-Seq data has been deposited at NCBI GEO under the accession GSE97767.

## Electronic supplementary material

Supplementary Information

Supplementary Tables
