# Supplementary material for: Genome and secretome analysis of Pochonia chlamydosporia provide new insight into egg-parasitic mechanisms
Source: Sci Rep. 2018 Jan 18;8:1123. doi: 10.1038/s41598-018-19169-5 (PMC5773674; doi:10.1038/s41598-018-19169-5)
Supplement: Supplementary file 1 — Supplementary Information [file 41598_2018_19169_MOESM1_ESM.pdf]

Genome and secretome analysis of *Pochonia chlamydosporia* provide new insight into egg-parasitic mechanisms

Runmao Lin[1,2,*], Feifei Qin[1,2,*], Baoming Shen[2,*], Qianqian Shi[2], Chichuan Liu[2], Xi Zhang[1,2], Yang Jiao[2], Jun Lu[1], Yaoyao Gao[1], Marta Suarez-Fernandez[3], Federico Lopez-Moya[3], Luis Vicente Lopez-Llorca[3], Gang Wang[2], Zhenchuan Mao[2], Jian Ling[2], Yuhong Yang[2], Xinyue Cheng[1,4] & Bingyan Xie[2,5]

Supplementary Figures

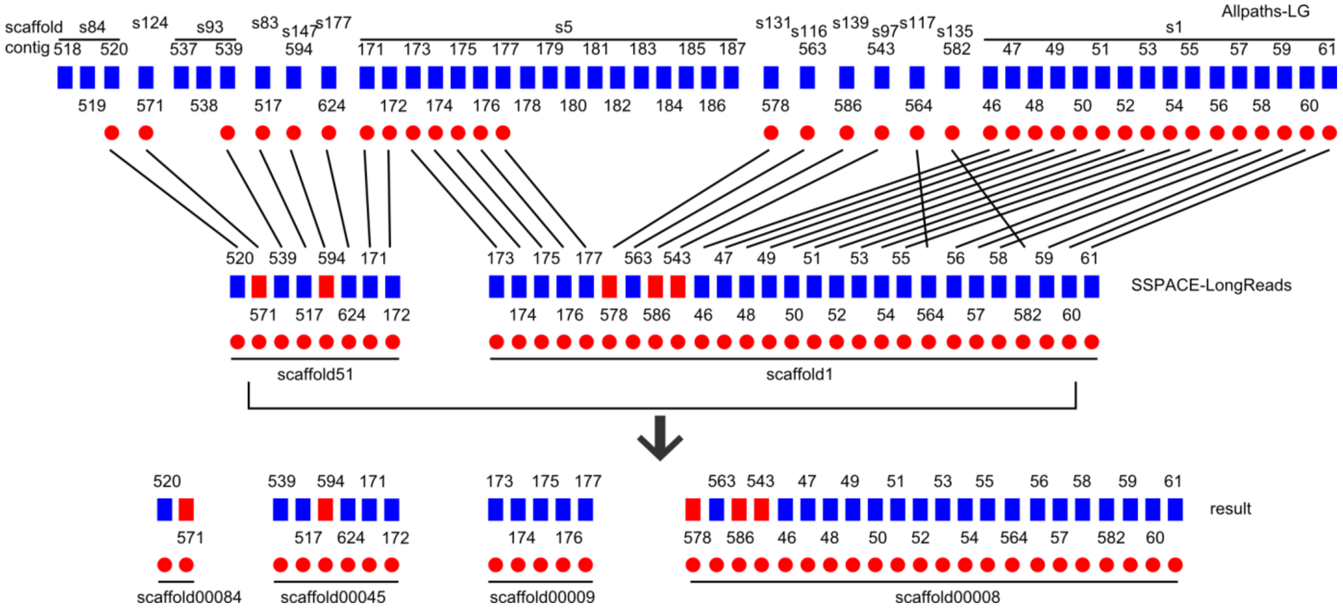

Supplementary Figure S1. Example of manually improved conflicts between Allpaths-LG and SSPACE-LongReads assemblies. contig520 (in scaffold s84) and contig571 (scaffold s124) in Allpaths-LG assembly could be joined to scaffold51 in SSPACE-LongReads assembly. But a conflict between two assemblies showed that three contigs (contig537, contig538 and contig539) were built as one scaffold (s93) in Allpaths-LG assembly, and contig571 and contig539 were built as one scaffold (scaffold51) in SSPACE-LongReads assembly. We manually broke the scaffold51 into two scaffolds (scaffold00084 and scaffold00045), one (scaffold00084) contained contig520 and contig571, and another (scaffold00045) contained contig539 and other contigs. Red and blue rectangles indicated “-” and “+” DNA strands, respectively.

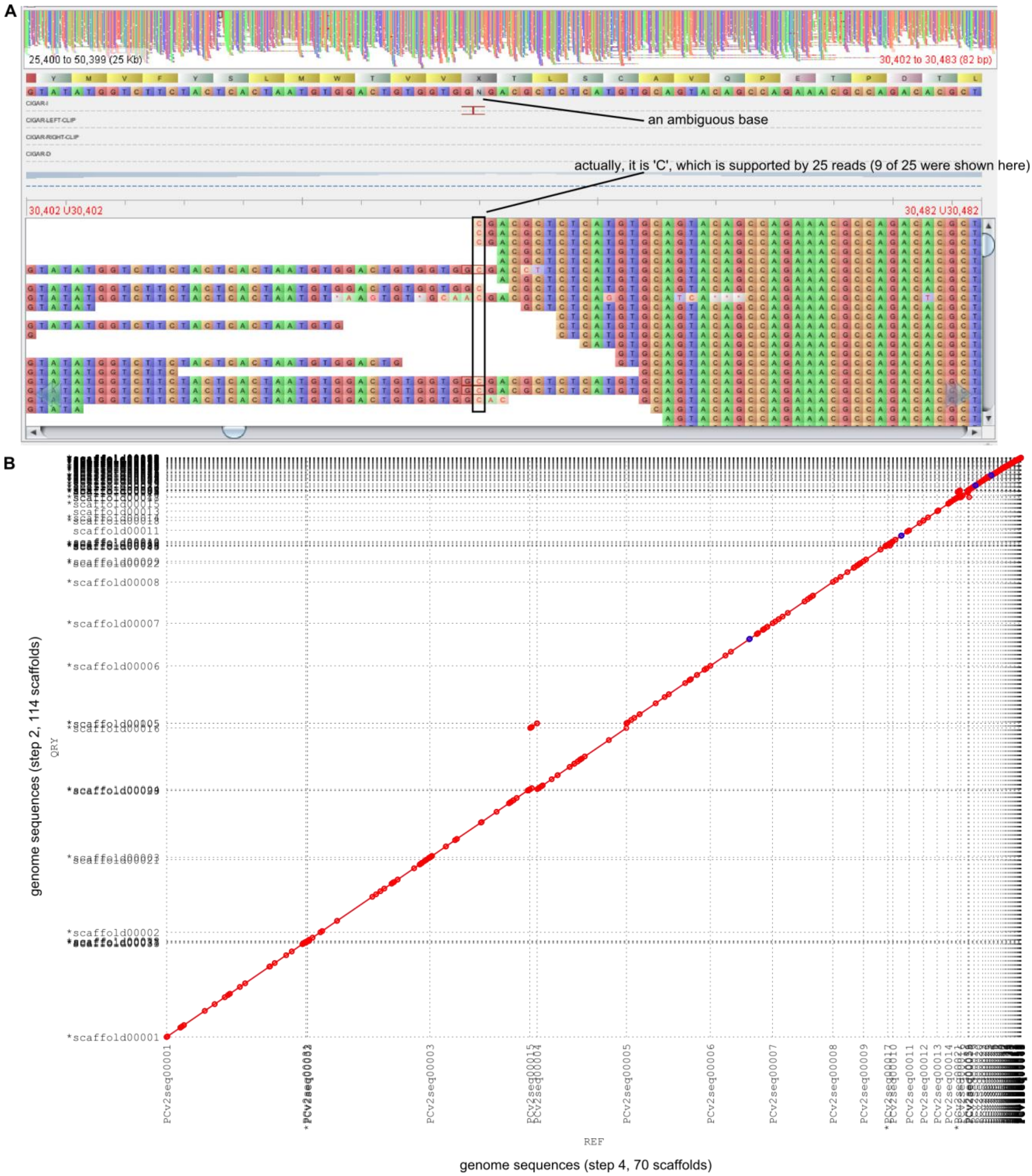

Supplementary Figure S2. Manually improved assembled sequences by Tablet viewer (A) and synteny between step 2 assembled sequences (114 scaffolds) and step 4 assembled results (70 scaffolds) (B). We aligned reads to assembled sequences using BWA software. As shown in (A), the previously assembled "N" in scaffold sequences is actually "C" by the view of reads alignments.

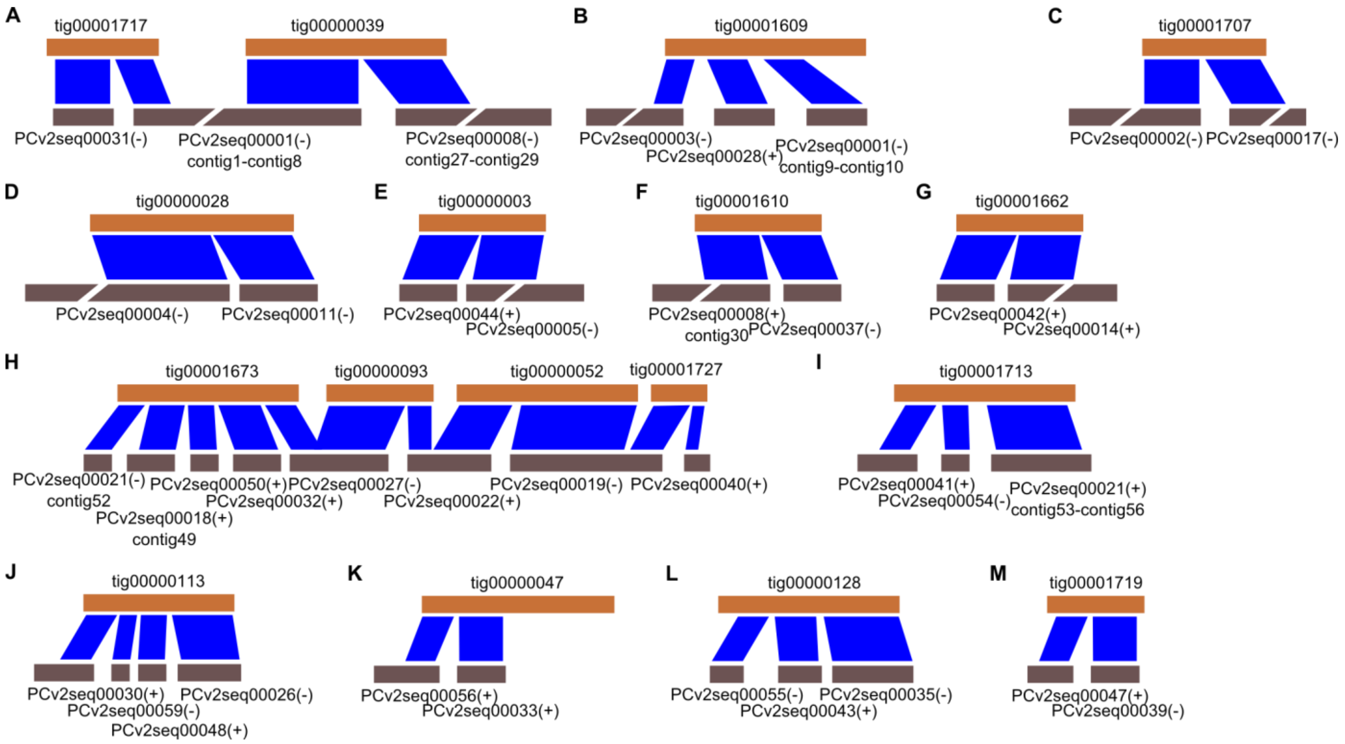

Supplementary Figure S3. Comparison between step4 assembly sequences and Canu assembly. A total of 35 scaffolds in step 4 assembly results could be further improved, such as: in (A), PCv2seq00031 (one scaffold), eight contigs (contig1–contig8 of scaffold PCv2seq00001), and three contigs (contig27–contig29 of PCv2seq00008) in step4 assembly results could be built as one super scaffold, which was supported by Canu assembled scaffolds (tig00001717 and tig00000039). “+” and “-” means forward and reverse DNA strands.

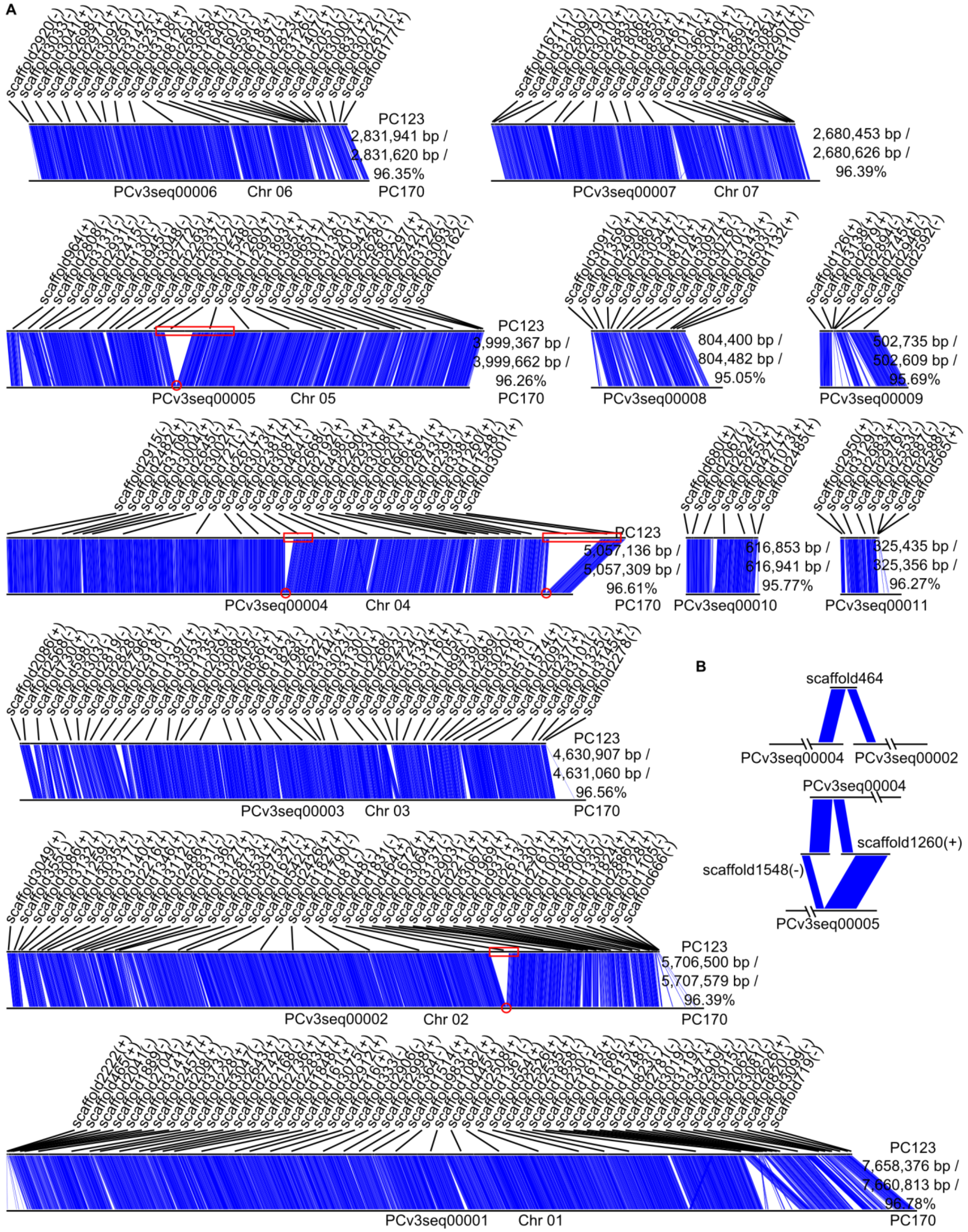

Supplementary Figure S4. Synteny between final assembled sequences of *Pochonia chlamydosporia* strain 170 (PC170) and reported sequences of *P. chlamydosporia* strain 123

(PC123). (A) Seven chromosome sequences of PC170 matched to 242 scaffolds of PC123. For example, 7,660,813 bp of PC170 Chr 01 sequences matched to 7,658,376 bp of PC123 sequences (53 scaffolds) with identity of 96.78%. "+" and "-" meant forward and reverse strands, respectively. **(B) Sequences of three scaffolds of PC123 each matched to two chromosomes of PC170.**

A

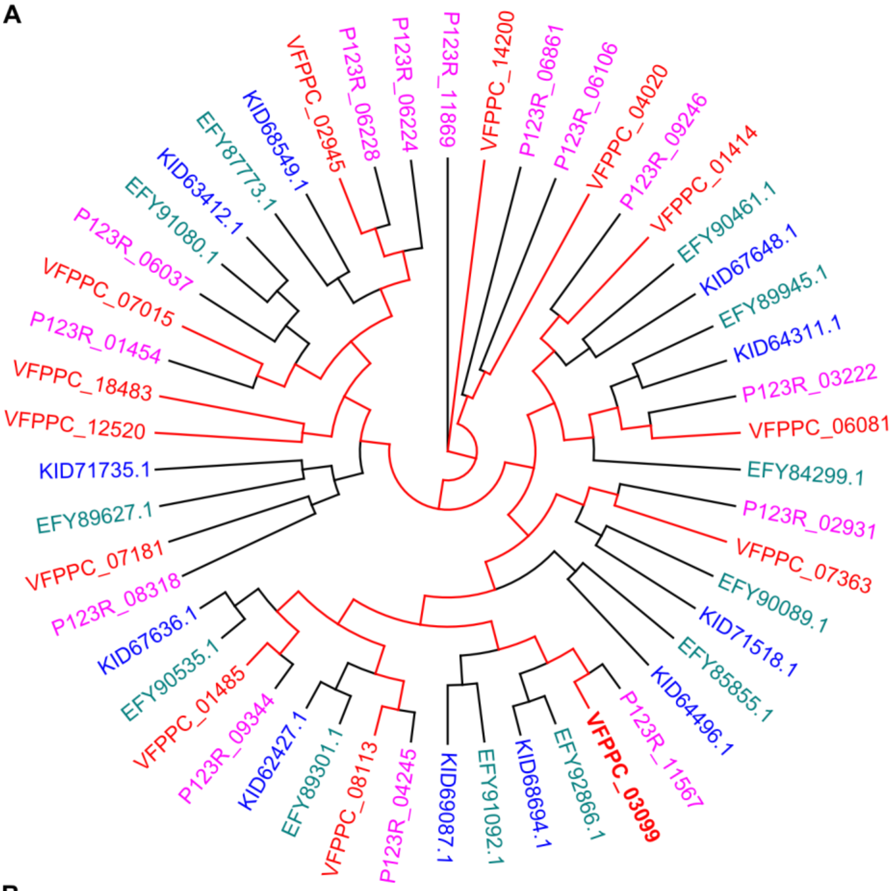
VFPPC_02945
P123R_06228
P123R_06224
P123R_11869
VFPPC_14200
P123R_06861
P123R_06106
VFPPC_04020
P123R_09246
VFPPC_01414
EFY90461.1
KID67648.1
EFY89945.1
KID64311.1
P123R_03222
VFPPC_06081
EFY84299.1
P123R_02931
VFPPC_07363
EFY90089.1
KID71518.1
EFY85855.1
KID64496.1
P123R_11567
VFPPC_03099
EFY92866.1
KID68694.1
EFY91092.1
KID69087.1
P123R_04245
VFPPC_08113
EFY89301.1
KID62427.1
P123R_09344
VFPPC_01485
EFY90535.1
KID67636.1
P123R_08318
VFPPC_07181
EFY89627.1
KID71735.1
VFPPC_12520
VFPPC_18483
P123R_01454
VFPPC_07015
P123R_06037
EFY91080.1
KID63412.1
EFY87773.1
KID68549.1

B

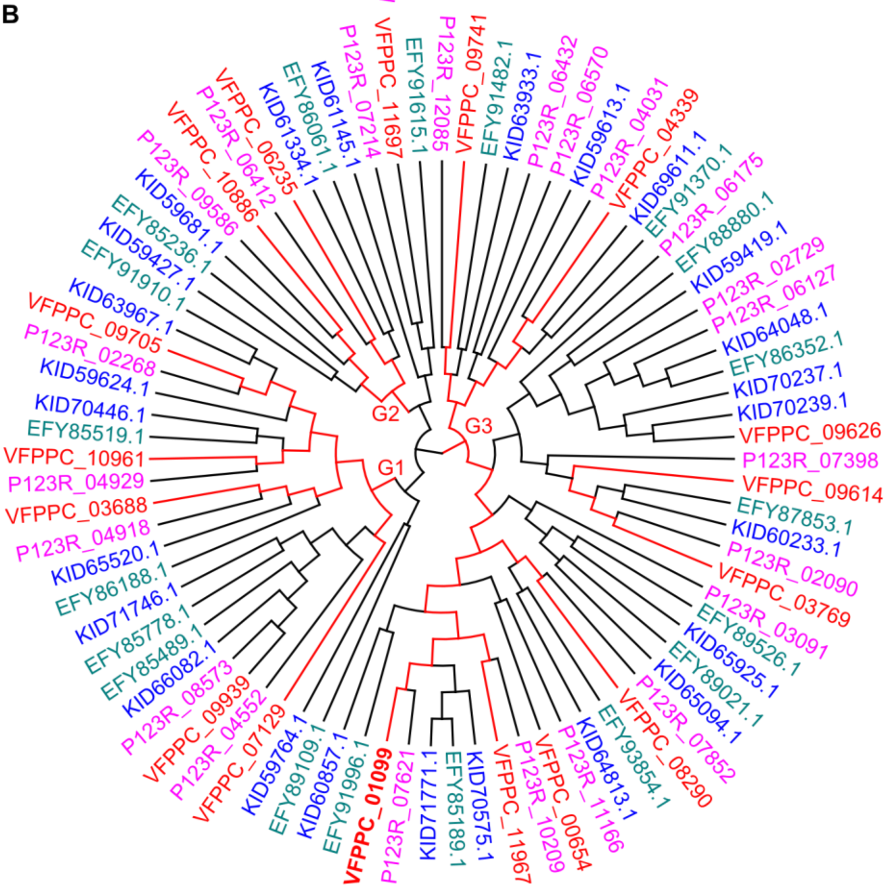
G1
G2
G3
P123R_09586
VFPPC_10886
P123R_06412
VFPPC_06235
KID61334.1
EFY86061.1
KID61145.1
P123R_07214
VFPPC_11697
EFY91615.1
P123R_12085
VFPPC_09741
EFY91482.1
KID63933.1
P123R_06432
P123R_06570
KID59613.1
P123R_04031
VFPPC_04339
KID69611.1
EFY91370.1
P123R_06175
EFY88880.1
KID59419.1
P123R_02729
P123R_06127
KID64048.1
EFY86352.1
KID70237.1
KID70239.1
VFPPC_09626
P123R_07398
VFPPC_09614
EFY87853.1
KID60233.1
P123R_02090
VFPPC_03769
P123R_03091
EFY89526.1
KID65925.1
EFY89021.1
KID65094.1
P123R_07852
VFPPC_08290
EFY93854.1
KID64813.1
P123R_11166
VFPPC_00654
P123R_10209
VFPPC_11967
KID70575.1
EFY85189.1
KID71771.1
P123R_07621
VFPPC_01099
EFY91996.1
KID60857.1
EFY89109.1
KID59764.1
VFPPC_07129
P123R_04552
VFPPC_09939
P123R_08573
KID66082.1
EFY85489.1
EFY85778.1
KID71746.1
EFY86188.1
KID65520.1
P123R_04918
VFPPC_03688
P123R_04929
VFPPC_10961
EFY85519.1
KID70446.1
KID59624.1
P123R_02268
VFPPC_09705
KID63967.1
EFY91910.1
KID59427.1
EFY85236.1
KID59681.1

C

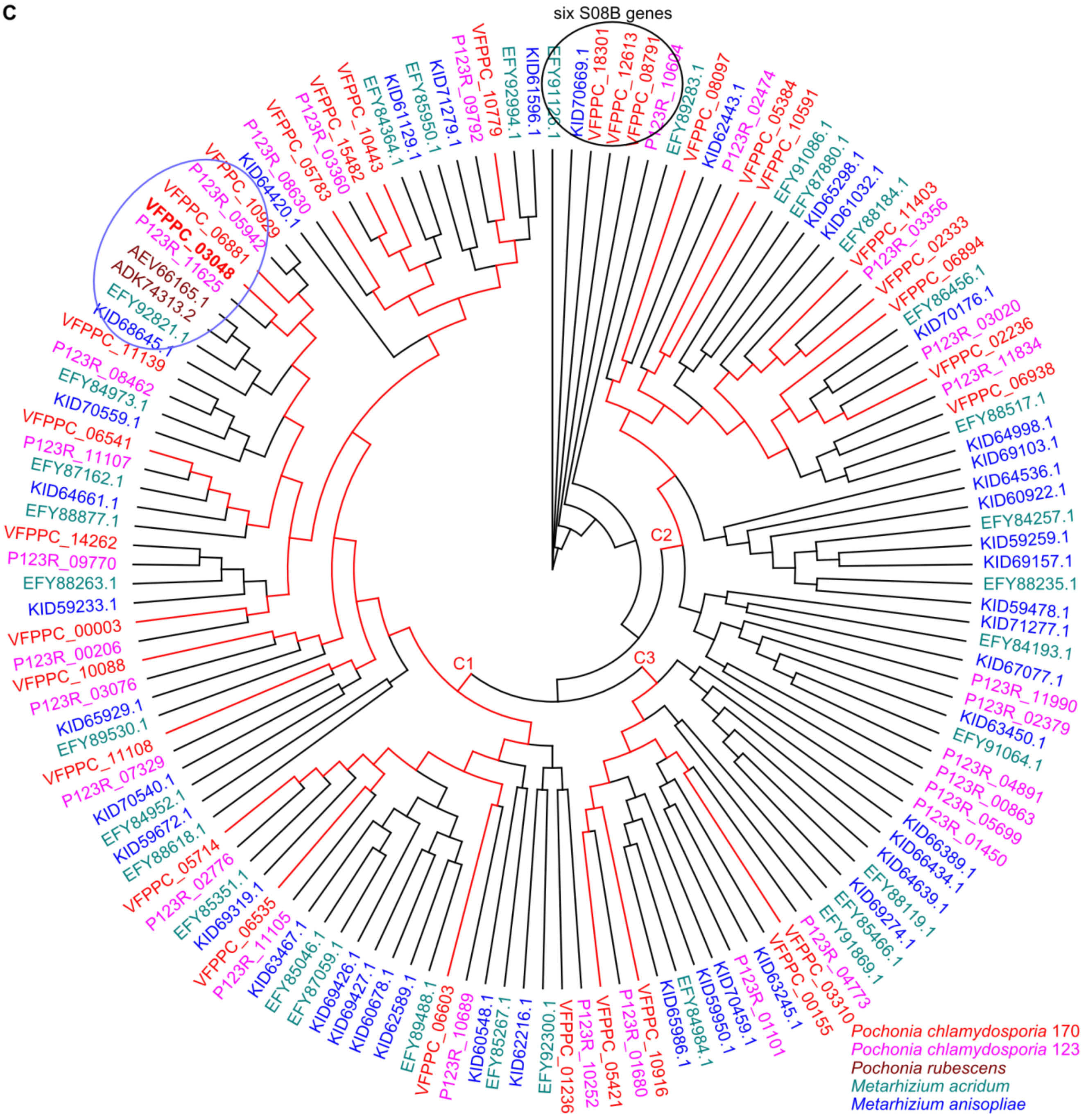

Supplementary Figure S5. Phylogenetic trees for S10 (A), GH18 (B), and S08A (C) genes from four fungal strains (PC170, PC123, *Metarhizium acridum*, and *Metarhizium anisopliae*) within the Clavicipitaceae Family. The domain sequences (Peptidase_S10: Serine carboxypeptidase; Peptidase_S8: Subtilase; Glyco_hydro_18: GH18) of these genes were annotated by Pfam analysis and were used to construct the phylogenies using PhyML with bootstrap value of 500. The best models for these S10, S08A, GH18 genes were WAG+I+G, WAG+I+G, and WAG+G+F, respectively, which were identified by ProtTest analyses. In these

trees, the phylogenetic relationships of PC170 genes were similar to their relationships in Figure 3D–F that were constructed by only PC170 gene sequences, and these conserved branches were marked in red color. In (C), two genes from *Pochonia rubescens* were included in the analysis and they were marked in the blue circle. Additionally, as six S08B genes being annotated by the GH18 domain from the Pfam analysis, they were included in the analysis and were marked in the black circle.

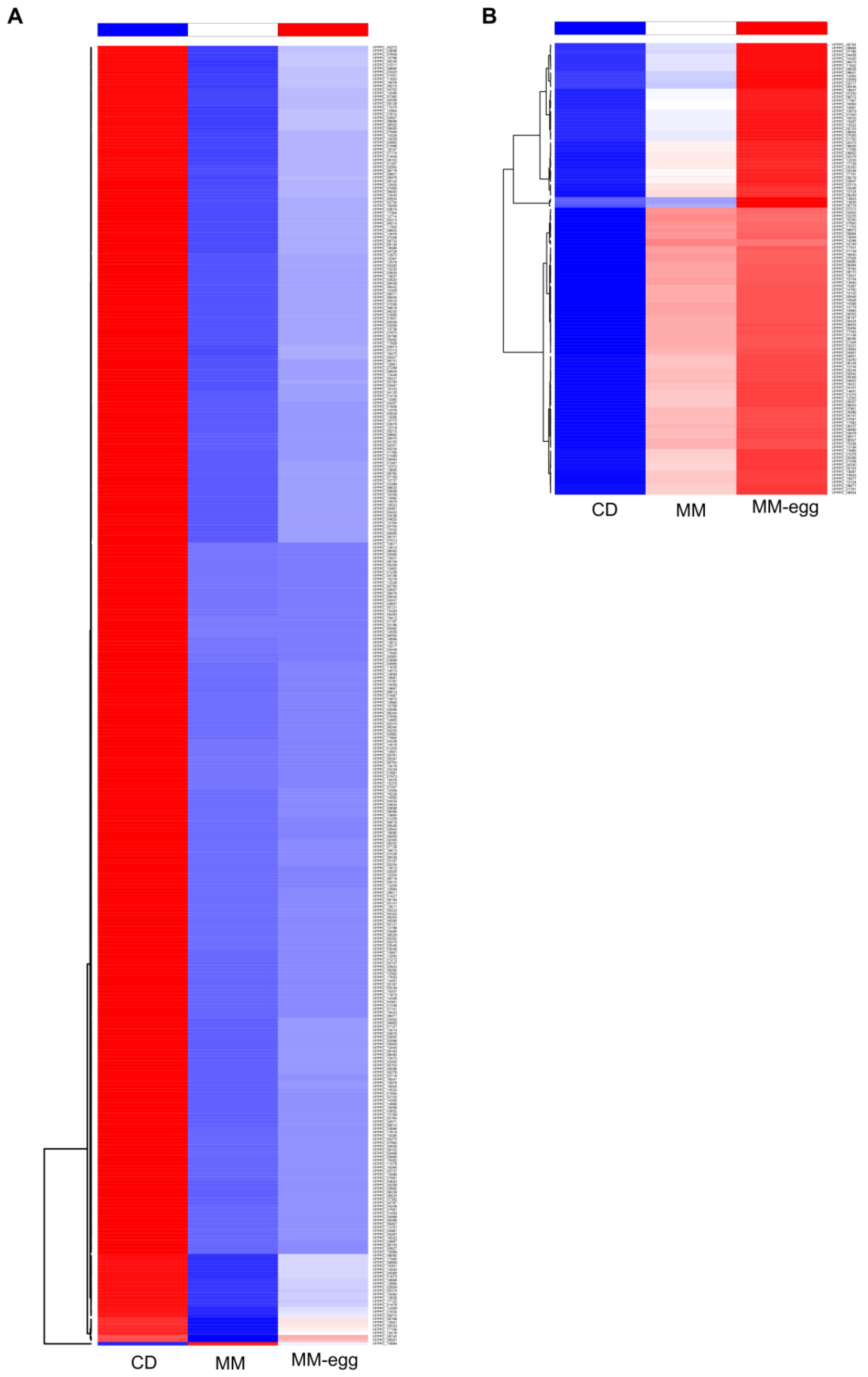

Supplementary Figure S6. Heatmaps for 369 and 129 genes with expression patterns of “CD>MM<MM-eggs” (A) and “CD<MM<MM-eggs” (B), respectively. They may be involved

in response to nutrient selection pressure and were listed in Supplementary Table S13. For the transcriptomic expression data, we used the “average” method to perform the hierarchical cluster analysis and then drew the heatmaps. During these processes, the “hclust” and “heatmap” functions in the R language (https://www.r-project.org/) were used to perform the analyses.

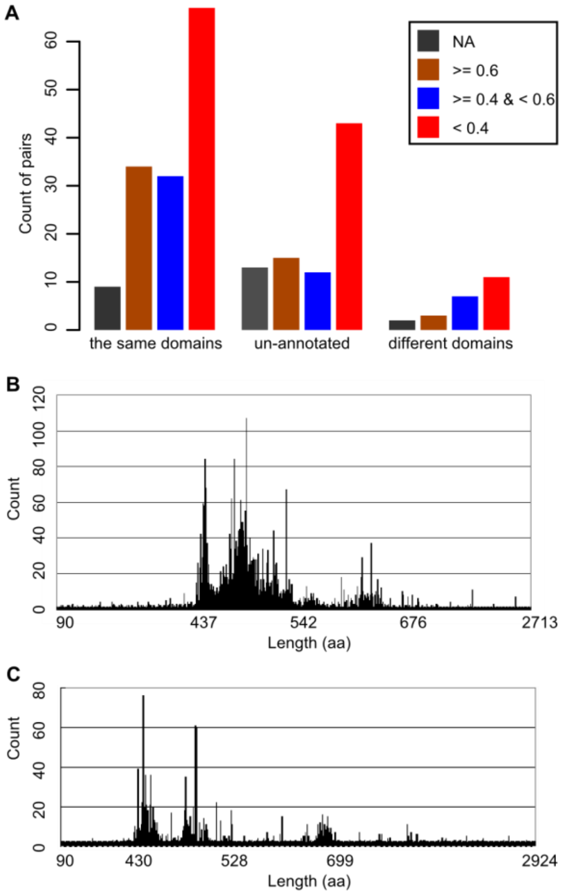

Supplementary Figure S7. Distribution of Pearson correlation coefficient values for 248 pairs of duplicated genes (A) and length distributions of homoglogous genes of two GH30 pair genes of VFPPC_07807-VFPPC_09315 genes (B) and VFPPC_01957-VFPPC_02227 (C), respectively.

A

```
>VFPPC_07807
MRAALLSTILASTGASAATLDRRAGASSWTSTANGQYKLSQFDAPVLGGSNPGIQDWDFKIFEKSKQKQTVKGFGACVTDSTVTAFNRVPSSARAQLFRD
LMTSDGINLNLMRHTIASSDLSGDPAYSYDDNNNQDDPNLNGFNLGDRGNAMASMLAEMRRLQGGMTLLGSPWSAPGWMKLNKKIMGTTDKNNLDPAHRQ
HYANYFVKYLQAYEKAGAHVDAVTLQNEPFNSRAQMPTMYVFPDESGQIIRDNLGPAIRNAGLNTKIWAWDHNTDVYSYPQTVFHTAPQFVNTAAWHCYA
GNDPNGWTPLTKFHNEFPNSEQYMTECWTATGTTDWKHSSNFALMPLQNWANGIIAWALGTYTGGGPSISGSDACHQCTGLVTVDPNSGSYTKTIDYYMM
GQFSKFMTKGGKVVDTSGSYLFNDNEGLEMVASVNPDGTRTVVIQNRYNHDIFVKVKAEAEGQPWGAKVPGTSVTTWVLPRA
>VFPPC_02227
MFLAILSSIIALANLSTVDADTVTTVDPSTWGVWEGWGVSLAWWAKAFGDREDLADALFTTNWQSFQGQSLPGLGFNIARYNAGASSTKAYNGSSMVVSP
NIKPSRQIDGFWLDWASSDPSSSSWDWSVDAKQRLMLQNARMKGADTFELFSNSPMWWMCYNHNPSGASDASKDNLQSWNYEQHAAYLANIDQYARQHWD
IDFQSVEAFNEPSSDYWSAMGGQEGCHFDVSTMDKVTAFLRTELSKRGSNSFIAASDETSYDAAVNTWKMMASASKDAIRRINVHGYQGGGGRRDQLYSL
AKQSNKVLWNSEYGDNDGSGKQMAANILLDFYWLHPTAWVYWQAVDIPGWGLIVGDNDAKTLDSVSTKYFVLAQFSRHIRTGMTILGGGNADTVAAYNAK
NKTLVVVAANWASAQYINFDLSRFTKTWKDGDLVKRWHTVVDGSENYAEHNDTFMQGSRFWSWFDTGSVQTFEINGVDL
```

B

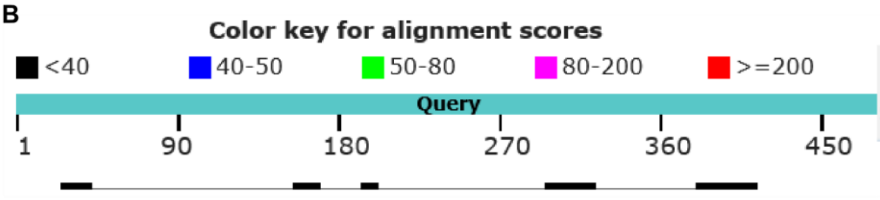

Max score	Total score	Query cover	E value	Ident
20.4	88.9	21%	0.14	28%

```
VFPPC_07807  380  GLVTVDPNSGSYTKTID-----YYMMGQFSKFMTKGGKVV  414
                  GL+  D ++    KT+D     Y+++ QFS+ +  G  ++
VFPPC_02227  351  GLIVGDNDA----KTLDSVSTKYFVLAQFSRHIRTGMTIL  386
```

C

```
VFPPC_07807      TVTAFNRVPSSARAQLFRDLMTSDGINLNLMRHTIASSDLSGDPAYSYDDNNNQDDPNLNGFNLGDRGNAMASMLAEMRRLQGGMTL
VFPPC_09315      TVIAFDRLSSRDYETVMRDLMTAEGINLSLMRHTIASSDLSLDPAYSYDDNDGKPDPNLSHFSIGQYGKNMTRMLRQMKHIQPEMVL
VFPPC_01957      TSLAWSGKRFGNRNDLADILFTRNDTQL-----------------------NGQTLPGM-GLNIARYNAGACSWNK--LSWDNDATM
VFPPC_02227      VSLAWWAKAFGDREDLADALFTTNWQSF-----------------------QGQSLPGL-GFNIARYNAGASS-----TKAYNGSSM
                 .  *:          :   *:* :  .:                       :.:  *.:  :.:.  .                  :
VFPPC_07807      LGSPWSAPGWMKLNKKIMGTTDKNNLDPAHRQHYANYFVKYLQAYEKAGAHVDAVTLQNEPFNSRAQMPTMYVFPDESGQIIRDNLG
VFPPC_09315      LGSPWSPPGWMQLDGVIMGSEVNNSLDHRYADAFARYFVKYIEAYAKAGVHVDAITLQNEPLANKAGMPSMYIGGNESAYLIRDHVG
VFPPC_01957      VESPNIDHSW-QMEG---------------------YWLDGISGDPATVSWNWSVDANQRAMLQKAKAR-----GADRFELFS----
VFPPC_02227      VVSPNIKPSR-QIDG---------------------FWLDWASSDPSSSSWDWSVDAKQRLMLQNARMK-----GADTFELFS----
                 : **.   .. :::                      :::.  ..   :  .  ::  ::  : ..*          :   ::
VFPPC_07807      PAIRNAGLNTKIWAWDHNTDV---------------------YSYPQTVFHTAPQFVNTAAWHCYAGN-DPNGWTPLTKFHNEFPNS
VFPPC_09315      PMLRKSRLQTEIWAFDHNTGETYLVHLDNKDTDFLTLYCEGDYEYPETVARVASDFVSASAWHCYEGNTEPGHWDPLTRFHDKFPHL
VFPPC_01957      --------NSPMW---------------------------------------------------WMCSNHN--PSGSNCIQP----ISGC
VFPPC_02227      --------NSPMW---------------------------------------------------WMCYNHN--PSG----------ASDA
                         :: :*                                                * *   *  *.            .
VFPPC_07807      EQYMTECWTATGTTDWKHSSNFALMPLQNWANGIIAWALGTYTGGGPSISGSDACHQCTGLVTVDPNSGSYTKTIDYYMMGQFSKFM
VFPPC_09315      DQYMTECYTARGHSDWIFTSTFTMQPLQNWANGIMAWALGTWTEGGPALAGSVPCHICTGLITVDPKTGSWTKEVDYYLIGQYSKFI
VFPPC_01957      NDENLDPKYQTQHAVYL--ATIAKYARDNW---------------GLTFESVEPFN--------EPSSHGWIG-----YMGPFEAWL
VFPPC_02227      SKDNLQSWNYEQHAAYL--ANIDQYARQHW---------------DIDFQSVEAFN--------EPSSDYW---------------
                 ..   :       : :   :.:   .  ::*               .  :  .  . :        :*.:  :
VFPPC_07807      TKG---GKVVDTSGSYLFNDNEGLEMVASVNPDGTRTVVIQNRYNHDIFV
VFPPC_09315      ARG---GHIVNSTGSHLDSSAMGIQAVASINPDGTRTVVIENRFNQDMWV
VFPPC_01957      AEKLGYGNWGRQEGCHINWDTMP-NVIEALRSE------LDNRGLHGTLV
VFPPC_02227      ------SAMGGQEGCHFDVSTMD-KVTAFLRTE------LSKRGSNSFIA
                       .      *.::  .    :    :..:      :.:*  :.  .
```

D

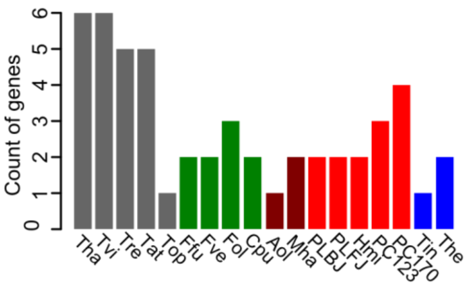

Tha, *Trichoderma harzianum*	Aol, *Arthrobotrys oligospora*
Tvi, *Trichoderma virens*	Mha, *Monacrosporium haptotylum*
Tre, *Trichoderma reesei*	PLBJ, *Purpureocillium lilacinum* PLBJ-1
Tat, *Trichoderma atroviride*	PLFJ, *Purpureocillium lilacinum* PLFJ-1
Top, *Tolypocladium ophioglossoides*	Hmi, *Hirsutella minnesotensis*
Ffu, *Fusarium fujikuroi*	PC123, *Pochonia chlamydosporia* 123
Fve, *Fusarium verticillioides*	PC170, *Pochonia chlamydosporia* 170
Fol, *Fusarium oxysporum*	Tin, *Tolypocladium inflatum*
Cpu, *Claviceps purpurea*	The, *Torrubiella hemipterigena*

Supplementary Figure S8. Comparison of two GH30 genes from two different duplicated

pairs. (A) Two genes representing two pair genes of VFPPC_07807-VFPPC_09315 and VFPPC_01957-VFPPC_02227 genes. (B) Aligned amino acid sequences of VFPPC_07807 against sequences of VFPPC_02227 using BLASTP server at NCBI. Low identity of 0.28 was generated and only five alignment blocks were found. **(C) Muscle alignment of GH30 domains from four GH30 genes in PC170.** Sequences of VFPPC_01957 and VFPPC_02227 belonged to PF14587 family; and sequences of VFPPC_07807 and VFPPC_09315 belonged to PF02055 family. (D) **Discovery of 51 homologous genes of *P. chlamydosporia* GH30 proteins in other fungal genomes.** One gene (HIM_08846) in *Hirsutella minnesotensis* with only 80 amino acids (aa) was excluded for phylogenetic analysis in Figure 5A, for its length was much less than that from other genes with hundreds of aa.

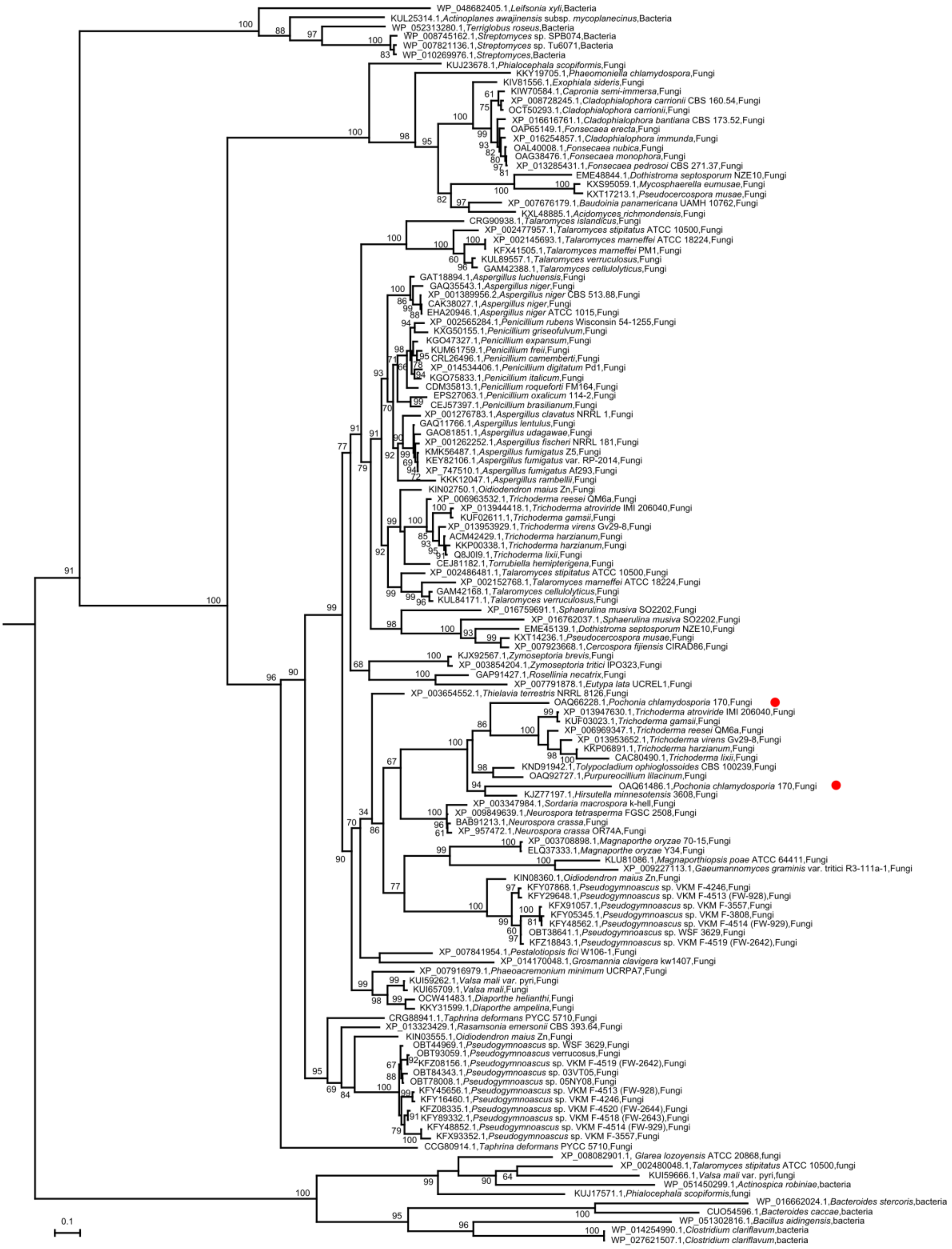

Supplementary Figure S9. The detailed of BF-D1 clade in Figure 4A. The two duplicated genes (VFPPC_07807 and VFPPC_09315) in *P. chlamydosporia* are clustered into a clade with some Hypocreales fungi, with VFPPC_07807 (accession number: OAQ66228) clustering closer to endophytic *Trichoderma* spp. and VFPPC_09315 (accession number: OAQ61486) clustering closer to nematode pathogens (*H. minnesotensis*). The support values of ≥ 60 are shown.

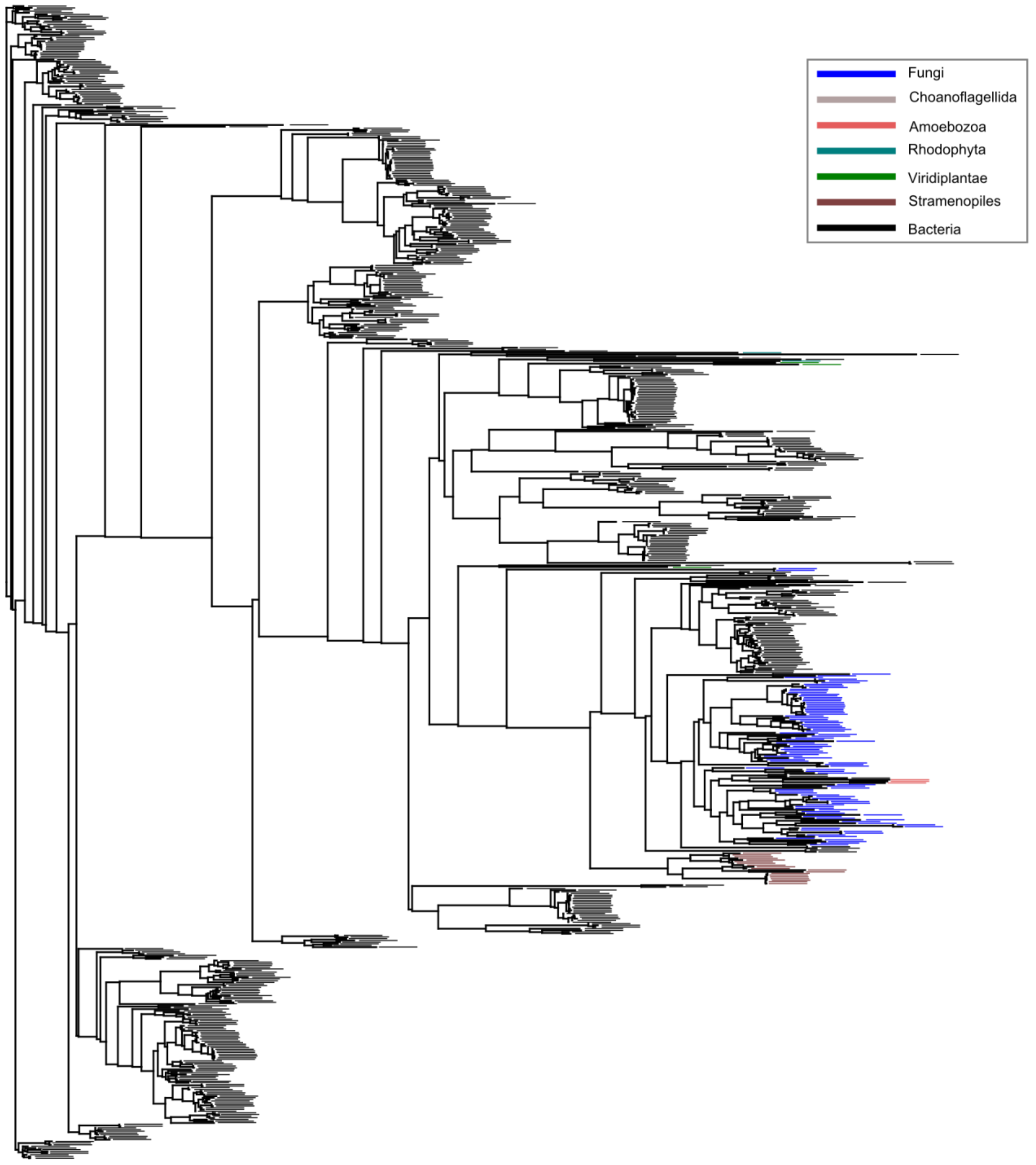

Supplementary Figure S10. Phylogeny of 689 homologous genes of VEFFC_02227 and VFPPC_01957 genes. The phylogeny was built based on GH30 domain (PF14587) sequences using FastTree.

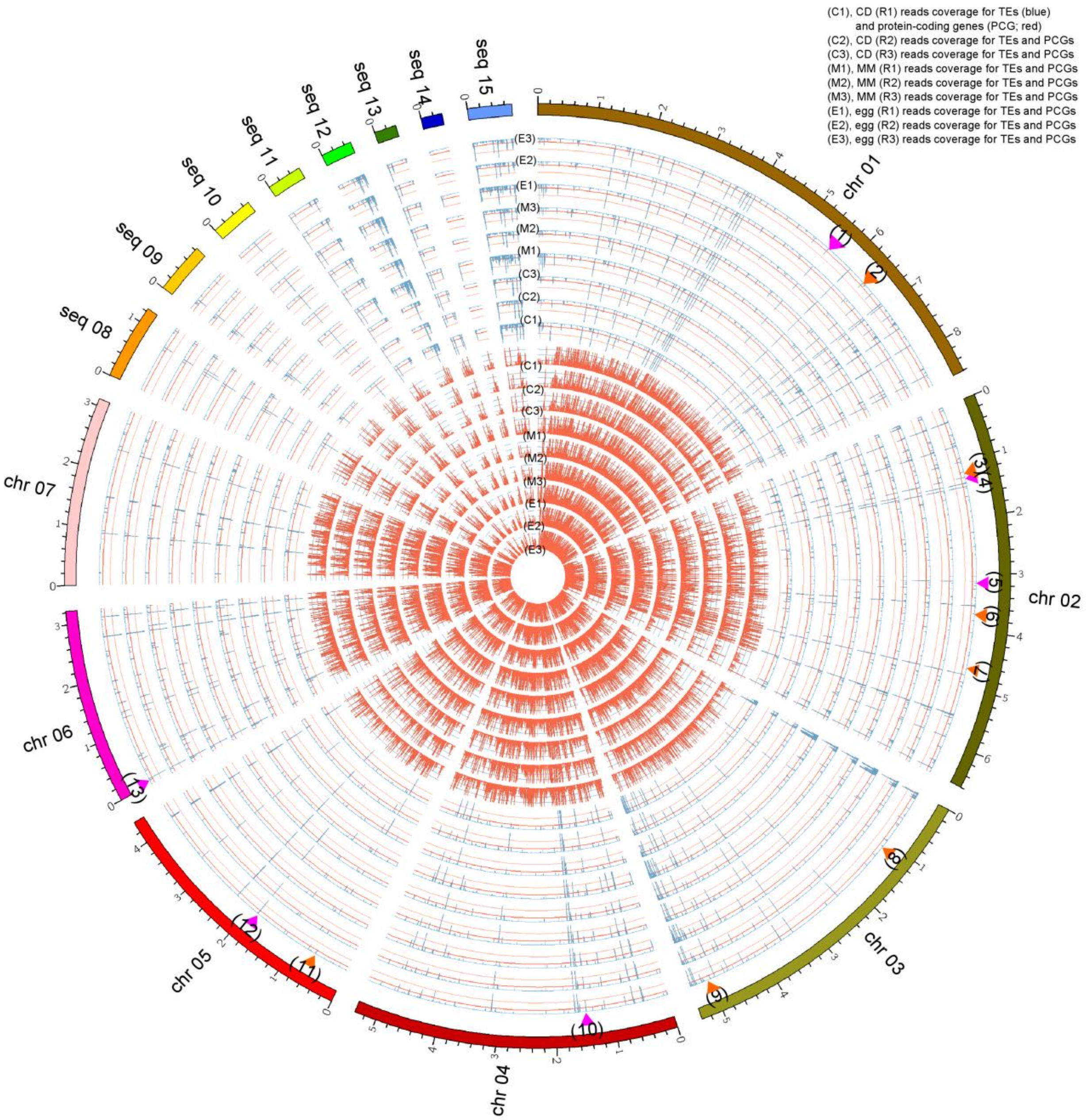

Supplementary Figure S11. Transcriptomic reads coverage of transposable elements (blue) and protein-coding genes (red). Three groups of transcriptome samples (CD, MM, and MM-egg) and each group with three replicates (R1, R2, and R3) were represented in the analysis. The RNA-seq data was aligned to genome sequences using Tophat software, which generated the alignment BAM files. To calculate the transcriptomic reads coverage of TEs and protein-coding genes, only unique mapping reads (i.e., one reads mapping to only one genomic region, which was indicated in the BAM files) were used for the coverage calculation. From the coverage peaks, we

identified 13 TE clusters representing by more covered reads from CD samples than from MM/MM-egg samples (orange triangles), or by more reads from MM/MM-egg samples than from CD samples (pink triangles). The detailed information of TEs and protein-coding genes for these 13 clusters were shown in Supplementary Table S18.

Supplementary Information

Genome assembly

Based on the Illumina sequencing reads from three libraries with average insert sizes of 165 bp, 760 bp and 4,261 bp (Supplementary Table S1), we used Allpaths-LG revision 42305[59] to assemble the *Pochonia chlamydosporia* strain 170 (PC170) nuclear genome sequence of 43.86 Mb (the step 1 results in Table 1) and a complete mitochondrial genome sequence[46]. The scaffold N50 and contig N50 of the Allpaths-LG asembly are 4,092 kb and 211 kb, and the GC content is 49.5% (Table 1). To obtain the chromosome sequences of PC170, we further sequenced the Single Molecule Real-Time (SMRT) sequencing data of Pacific Biosciences (PacBio) RS long reads (Supplementary Table S1) to improve the assembly. To correct error bases of these long reads, we used LoRDEC version 0.5 method[60] to analyze the data with the 165 bp and 760 bp libraries Illumina reads. To improve the scaffold building, the PacBio long reads were aligned to contig sequences from Allpaths-LG assembly using BLASR[89], and the matched information was used to rebuild scaffolds using SSPACE-LongRead version 1.1[61]. A number of short scaffolds each containing one contig in Allpaths-LG results were joined with other scaffolds by PacBio long reads, such as four scaffolds (s131 (contig578), s116 (contig563), s139 (contig586) and s97 (contig543)) with length of smaller than 8 kb in Allpaths-LG assembly forming the new scaffold1 with other sequences in SSPACE-LongRead results (Supplementary Figure S1), supporting by long reads ranging from 3 to 70. However, conflicts of scaffold results were found between Allpaths-LG and SSPACE-LongRead assemblies, for example: 17 contigs (contig171-contig187) were joined as one scaffold of s5 in Allpaths-LG method, but 7 of these 17 contigs (contig171-contig177) were joined with other contigs to form scaffold1 in SSPACE-LongRead results (Supplementary Figure S1). To avoid false assembly, we broke the previous scaffolds and used consensus assembled segments in two methods to build new scaffolds, as shown in Supplementary Figure S1, 7 contigs (contig171-contig177) forming one scaffold and other contigs forming another scaffold. And we obtained 114 scaffolds, the step 2 results in Table 1.

We also performed *de novo* assembly of LoRDEC corrected PacBio RS long reads using Canu version 1.2[49], which generated 103 scaffolds with the total length of 45.26 Mb. And the assembled

sequences were improved, mainly of “fix break” process at several genomic regions by performing Pilon program veresion 1.17[62] analysis. A comparison analysis of genomic sequences between hybrid assembly (Allpaths-LG and SSPACE-LongRead methods, the step 2 results) and Canu assembly by MUMmer version 3.23[90] analysis showed the high level of synteny for these sequences. As randem errors exist in PacBio SMRT sequencing[91], we presumed the hybrid assembly was much more correct than the Canu assembly. Therefore, we used Canu and PacBio RS long reads to improved the hybrid assembly, including filling gaps and joining scaffolds.

We split the Canu assembly into sequence segments with the maximum length of 100 kb and use these segments to improve the genome sequences of step2 results by performing Jelly program[63] implemented in PBSuite version 15.8.24 (https://sourceforge.net/projects/pb-jelly/), which obtained 100 scaffolds. The results were improved by Pilon analysis, filling 20 gaps and generating the step 3 results in Table 1. Then we used LoRDEC corrected PacBio RS long reads to further improve these 100 sequences by PBSuite Jelly program, and we got a total of 72 scaffolds (203 contigs). Compared to step2 results, 42 scaffolds had been reduced by Canu assembled sequences and corrected long reads. To confirm the sequences of filled gap regions, we used Pilon to improve the sequences based on Illumina reads, and used Tablet viewer[64] to check the reads alignments (Supplementary Figure S2A). Pilon analysis improved 2,369 regions of the assembly and filled 47 gaps; and our manual improving format using Tablet viewer filled 40 gaps and fixed hundreds of ambiguous bases (Supplementary Figure S2A; the number of continuous ‘N’s < 10 in the contig sequences; and runs of 10 or more ‘N’s are estimated gaps), which obtained 72 scaffolds (116 contigs). And we found that two scaffolds (3,638 bp and 1,341 bp) were completely aligned to other two scaffolds (136,565 bp and 97,483 bp) respectively. Therefore, we removed these two short scaffolds (3,638 bp and 1,341 bp) and finally got the 70 scaffolds, the step 4 results (Table 1). The high synteny between step 2 results and step 4 results was identified (Supplementary Figure S2B) by MUMmer analysis.

And we aligned the sequences of step 4 results against Canu assembly using BLASTN (1e-5) and found that some scaffolds could be further improved (Supplementary Figure S3), such as three scaffolds (PCv2seq00031, PCv2seq00001 and PCv2seq00008) were actually one super-scaffold (Supplementary Figure S3A) and PCv2seq00001 that includes 10 contigs was actually belonging to two super-scaffolds (Supplementary Figure S3AB). We manually improved the

scaffold-building of these 35 scaffolds by using 100 Ns to join scaffolds. Finally, we obtained 49 scaffolds of PC170 (Table 1). And we found the high sequence similarity between PC170 and *P. chlamydosporia* strain 123 (PC123)[12] genomes by BLASTN (Supplementary Figure S4; Supplementary Table S5). For the differences in Supplementary Figure S4B, the sequencing raw data supported the accuracy of PC170 assembly, which was viewed by Tablet viewer.

Discovery of centromere proteins and telomere regions

In total, 11 centromere proteins are discovered, with 7 and 8, respectively, determined by Pfam annotation and homology-based methods by searching sequences against reported genes (Figure 1A; Supplementary Table S2)[24]. Telomere sequences with high similarity to the *N. crassa* VR telomere region genomic sequences represented by TTAGGG tandem repeats are identified at the ends of 10 scaffolds (Figure 1A; Supplementary Table S3)[25]; they have also been found in the telomeric regions of Pezizomycotina fungi[26]. In our analysis with Tandem Repeats Finder[27], we find TTAGGG repeats at the left ends of two scaffolds (PCv3seq00002 and PCv3seq00013) and the right ends of six scaffolds (PCv3seq00001, PCv3seq00004, PCv3seq00007, PCv3seq00010, PCv3seq00017, and PCv3seq00030) (Supplementary Table S4), indicating that the telomere regions of the chromosomes are involved.

Discovery of repeat sequences

We identified transposon elements (TEs) sequences following the previous pipeline[33,66], with *de novo* and homology-based detection methods. For the *de novo* methods, Piler[92], RepeatScout version 1.0.5[93], and RECON version 1.08[94] were used to identified 79, 242, and 62 TE sequence families in the genome sequences, respectively. Based on these TE families, We used RepeatMasker version 4.0.5 to scan the whole-genome sequences to discover TE sequences. For the homology-based method, we used RepeatMasker to identified TE sequences based on the sequence families from Repbase version 19.06[95]. Finally, we discovered TE sequences occupying about 7.19% of genome sequences (Supplementary Table S6). We applied the same methods to identified TE sequences in PC123 genome and found about 1.20% were TEs (Supplementary Table S6).

Additionally, we used Tandem Repeat Finder version 407b[27] with parameters of “2 7 7 80 10

50 500 -f -d -m" to identified tandem repeats in PC170 genome sequences. The analysis identified (TTAGGG)n sequences at the left ends of PCv3seq00002 and PCv3seq00013, and the right ends of PCv3seq00001, PCv3seq00004, PCv3seq00007, PCv3seq00010, PCv3seq00017, and PCv3seq00030 (Supplementary Table S4). As previous reported telomeric regions in Pezizomycotina fungi representing patterns of (TTAGGG)n sequences[26], the discovery of TTAGGG repeats at the end of eight scaffolds would help to determine telomeres.

Gene prediction and functional annotations

We predicted the protein-coding genes using the previous methods[33]. Our previous phylogenetic analysis of *P. chlamydosporia* and other species in Hypocreales based on mitochondrial genomes indicated their phylogenetic relationships[46,47]. In Augustus version 2.7 software[96], the training annotation files of several fungi species had been published. And we choose the "fusarium_graminearum" training file to predict 12,434 genes. The GeneMark-ES version 2.3f method[97] identified 13,769 genes. Both Augustus and GeneMark-ES methods identified 11,782 genes, and we predicted another 2,422 genes using Eugene version 4.1[98] as the method describing in previous research[66], which integrated transcription start sites, exon junctions, transcriptome and homologous protein information. The transcription start sites were predicted using Netstart v1.0c[99], and exon junctions were identified using Tophat version 2.0.13[100]. The transcriptome sequences were assembled using IDBA-tran version 1.1.1[101]. And homologous proteins downloaded from the Swiss-Prot database version 2015-07-22[102] were used for gene prediction.

To annotate the function of these 14,204 genes, we employed several methods including homologous proteins annotations by searching amino acid (aa) sequences against NCBI fungi refseq sequences version 2015-07-10 and Swiss-Prot database version 2015-07-22 using BLASTP (E-value of 1e-5), domain annotations by search aa sequences against Pfam version 27.0 using HMMER (http://hmmer.janelia.org/). Additionally, we explored Gene Ontology (GO) annotations using BLAST2GO[103], the euKaryotic Clusters of Orthologous Groups (KOG) annotations using BLASTP (E-value of 1e-5), and candidate pathogenic factor annotations based on sequences from the Pathogen-Host Interactions (PHI) database[73] using BLASTP (E-value of 1e-50).

To identify CAZymes and proteases, we uploaded the protein sequences to three web servers to annotate the genes, including CAT[74], dbCAN[75], and MEROPS[76]. For genes belonging to GH18

family chitinases, S08A family and S10 family proteases, three proteins were reported to involved in pathogenesis[14,15] (Supplementary Table S12). We carefully checked the PHI database annotations for proteins belonging to these three families, and identified other five genes (S08A: VFPPC_06603 and VFPPC_10779; GH18: VFPPC_03688, VFPPC_07129, and VFPPC_08586) annotated by PHI database with E-value cut-off of 5e-23.

Additionally, we identified secreted proteins following the previous methods[33]. We identified one secreted protein if it contained signal peptides detected by at least two algorithms among SignalP version 4.0[67], TargetP version 1.1[68], Phobious version 101[69], and Predisi[70], and it did not contain transmembrane sequences detected by at least one of the methods among SignalP, Phobious and TMHMM, version 2.0c[71]. We used the same pipeline to identified secreted proteins of other seven fungal strains (Supplementary Table S9). The PC123 gene sets were downloaded from the fungalinteractions website (http://www.fungalinteractions.org/index.php/genoma). In this study, we used the predicted nucleic acid sequences for 12,122 genes from PC123 (the file name of “Pc-11079-transcripts-AUG.fasta” on the fungalinteractions website) and translated these sequences to protein sequences for further analysis. When compared the secreted proteins of PC170 and PC123, we identified one pair of orthologous genes (VFPPC_03099 and P123R_11567) having different prediction, for no signal peptide discovered for VFPPC_03099, but P123R_11567 being identified as a secreted protein. We used InParanoid algorithm version 4.1[104] to further confirm that they were orthologous genes with 100% confidence score. Therefore, we considered VFPPC_03099 as a secreted protein in this study.

Orthologous groups

To investigate orthologous groups of seven fungal species including *P. chlamydosporia* 170, *M. acridum*, *U. virens*, *B. bassiana*, *P. lilacinum*, *T. reesei*, and *F. oxysporum* (Supplementary Table S11), we performed analysis using OrthoFinder version 0.7.1[78] based on their protein sequences. The analysis identified 23,315 groups, including 3,810 single-copy gene groups (i.e., 1:1 genes among species in a group) and 13,341 single gene groups (i.e., only one gene in a group). To identified orthologous genes between *P. chlamydosporia* 170 and *P. chlamydosporia* 123, we performed Inparanoid analysis with the confident scores cut-off of 0.5.

Transcriptome analysis

The fungus PC170 was originally isolated from root-knot nematode *Meloidogyne incognita* eggs. We prepared samples for transcriptome sequencing as previous descriptions in study by Rosso et al.[11], which investigated the transcriptome using the cDNA-amplified fragment length polymorphism approach without whole genomic data. The strain was grown on potato dextrose agar (PDA) at 28°C for seven days and conidia were harvested. Three media were prepared for subculture, including nutrient rich medium (CD: 30 g sucrose, 3 g $NaNO_3$, 0.5 g $MgSO_4$, 0.5 g KCl, 1.0 g K_2HPO_4 , 0.01 g $FeSO_4$) l^{-1}, minimal medium (MM: 1 mg sucrose, 14 mg $NaNO_3$, 0.25 g $MgSO_4$, 0.25 g KCl, 0.5 g K 2 HPO_4 , 0.06 g $FeSO_4$) l^{-1}, and minimal medium with RKN eggs (MM-egg). Three replicates (three groups: R1, R2, and R3) were taken from all three treatments (CD, MM, and MM-egg) at the time-point when eggs were added (referred as 0 hour), and time-points at 2, 4, 8 hours were taken, which were immediately frozen in liquid nitrogen. For each treatment, time-points were pooled to exact total RNA which was carried out using the RNeasy Plant Mini Kit (Qiagen) and was performed according to the manufacturer's instructions. And the total RNA was sequenced using Illumina Hiseq 2000 at Berry Genomics Co., Ltd (Beijing, China). We analyzed the transcriptome and calculated the gene expression fragments per transcript kilobase per million fragments mapped (FPKM) using Cufflinks version 2.2.1[105] based on the Tophat version 2.0.13[100] analysis, which was analyzed following reported protocols in study by Trapnell et al.[86]. If two duplicated genes had a Pearson correlation coefficient value less than 0.4 as calculated based on the transcriptome expression data, they were defined to show a weak correlation, as previously described[106].

Real-time analysis

The samples and RNA isolation were prepared as shown above (in the "Transcriptome analysis" section). And beta-tubulin (reverse 5'-CCATTCGACAAAGTAGGTCGAGTT-3', forward 5'-TCCCTCGTCTGCACTTCTTCA-3') expression profiles were initially used to validate and determine the most appropriate sampling times for differential gene expression. Gene-specific primers were designed based on the sequences of the selected gene (Supplementary Table S19). Transcript levels were measured using TaKaRa SYBR Premix Ex Taq II. The thermal profile was performed in a Biorad CFX manager, one cycle at 94°C for 30s and 40 cycles at 95°C for 30 s, at

specific annealing temperatures for 15 s and at 72°C for 15 s.

Phylogenetic analysis

When performing phylogenic analysis of proteases, we found one gene (VFPPC_10429) belonging to S10 family had 239 amino acids (aa), which was at least 241 aa shorter than other S10 genes. Although sequences of VFPPC_10429 (from 143 to 238 aa) matched to S10 sequence (MER0000412) in MEROPS database (E-value: 6.60e-05; Identity: 0.29), it could not be annotated by Peptidase_S10 (PF00450.17; Serine carboxypeptidase) domain in Pfam and Peptidase_S10 domain in NCBI Conserved Domain Database[107]. Thus, we did not use this gene for S10 gene family phylogenic analysis (Figure 2D).

To identified the best models for S10, S08A, and GH18 gene phylogenetic analyses, we analyzed the sequences using ProtTest version 3.4[80], which indicated WAG+I+G model for S10 and S08A genes and WAG+I+G+F model for GH18 genes, supporting by both the Akaike Information Criterion (AIC)[108] and the Bayesian Information Criterion (BIC)[109] methods.

To investigate the evolutionary history of one duplicated GH30 gene pair (VFPPC_02227-VFPPC_01957), we used the similar method that analyzed the evolution of another duplicated GH30 gene pair (VFPPC_07807-VFPPC_09315) as shown in "Materials and methods" section. We deposited the aa sequence of the PC170 gene (VFPPC_01957) in the NCBI nr database (E-value threshold of 1e-5) for alignment analysis, and found 1,651 homologous sequences, including 1,307 (79.16%) genes from bacteria and 344 (20.84%) genes from eukaryotes. Their lengths ranged from 90 to 2,924 aa, with the majority from 430 to 528 aa (944, 57.18%) (Supplementary Figure S7C). Most of the sequences (933) encoded PF14587 domains (GH30) with lengths ranging from 84 to 391 aa. To avoid the presence of too many gaps for alignment, we used 689 GH30 domain (Pfam accession: PF14587) sequences ranging from 180 to 260 aa to build phylogeny, including 562 (81.57%) from bacteria and 127 (18.43%) from eukaryotes (Supplementary Figure S10; Supplementary Table S17).

References

89. Chaisson, M. J. & Tesler, G. Mapping single molecule sequencing reads using basic local alignment with successive refinement (BLASR): application and theory. *BMC Bioinformatics.*

13, 238 (2012).

90. Kurtz, S. *et al.* Versatile and open software for comparing large genomes. *Genome Biol.* **5**, R12 (2004).

91. Ross, M. G. *et al.* Characterizing and measuring bias in sequence data. *Genome Biol.* **14**, R51 (2013).

92. Edgar, R. C. & Myers, E. W. PILER: identification and classification of genomic repeats. *Bioinformatics.* **21**, i152-8 (2005).

93. Price, A. L., Jones, N. C. & Pevzner, P. A. De novo identification of repeat families in large genomes. *Bioinformatics.* **21**, i351-8 (2005).

94. Bao, Z. & Eddy, S. R. Automated de novo identification of repeat sequence families in sequenced genomes. *Genome Res.* **12**, 1269-76 (2002).

95. Bao, W., Kojima, K. K. & Kohany, O. Repbase Update, a database of repetitive elements in eukaryotic genomes. *Mob. DNA.* **6**, 11 (2015).

96. Stanke, M., Keller, O., Gunduz, I., Hayes, A., Waack, S. & Morgenstern, B. AUGUSTUS: ab initio prediction of alternative transcripts. *Nucleic Acids Res.* **34**, W435-9 (2006).

97. Ter-Hovhannisyan, V., Lomsadze, A., Chernoff, Y. O. & Borodovsky, M. Gene prediction in novel fungal genomes using an ab initio algorithm with unsupervised training. *Genome Res.* **18**, 1979-90 (2008).

98. Schiex, T., Moisan, A. & Rouzé, P. EuGene: an eukaryotic gene finder that combines several sources of evidence. *Comput. Biol.* **2066**, 111-125 (2001).

99. Pedersen, A. G. & Nielsen, H. Neural network prediction of translation initiation sites in eukaryotes: perspectives for EST and genome analysis. *Proc. Int. Conf. Intell. Syst. Mol. Biol.* **5**, 226-33 (1997).

100. Trapnell, C., Pachter, L. & Salzberg, S. L. TopHat: discovering splice junctions with RNA-Seq. *Bioinformatics.* **25**, 1105-11 (2009).

101. Peng, Y., Leung, H. C., Yiu, S. M., Lv, M. J., Zhu, X. G. & Chin, F. Y. IDBA-tran: a more robust de novo de Bruijn graph assembler for transcriptomes with uneven expression levels. *Bioinformatics.* **29**, i326-34 (2013).

102. UniProt Consortium. UniProt: a hub for protein information. *Nucleic Acids Res.* **43**, D204-12 (2015).

103. Conesa, A., Götz, S., García-Gómez, J. M., Terol, J., Talón, M. & Robles, M. Blast2GO: a universal tool for annotation, visualization and analysis in functional genomics research. *Bioinformatics.* **21**, 3674-6 (2005).

104. Ostlund, G. *et al.* InParanoid 7: new algorithms and tools for eukaryotic orthology analysis. *Nucleic Acids Res.* **38**, D196-203 (2010).

105. Trapnell, C. *et al.* Transcript assembly and quantification by RNA-Seq reveals unannotated transcripts and isoform switching during cell differentiation. *Nat. Biotechnol.* **28**, 511-5 (2010).

106. Evans JD. Straightforward statistics for the behavioral sciences. Pacific Grove, Calif: Brooks/Cole Publishing; 1996.

107. Marchler-Bauer, A. *et al.* CDD: NCBI's conserved domain database. *Nucleic Acids Res.* **43**, D222-6 (2015).

108. Akaike, H. In *Second international symposium on information theory*. Information theory and an extension of the maximum likelihood principle. Akademinai Kiado, pp:267–281 (1973).

109. Schwarz G. Estimating the dimension of a model. *Annals of Statistics.* **6**, 461-464 (1978).
